# Interpenetrated and Bridged Nanocylinders from Self-Assembled Star Block Copolymers

**DOI:** 10.1021/acs.macromol.3c02088

**Published:** 2024-01-30

**Authors:** Esmaeel Moghimi, Iurii Chubak, Konstantinos Ntetsikas, Georgios Polymeropoulos, Xin Wang, Consiglia Carillo, Antonia Statt, Luca Cipelletti, Kell Mortensen, Nikos Hadjichristidis, Athanassios Z. Panagiotopoulos, Christos N. Likos, Dimitris Vlassopoulos

**Affiliations:** † Institute of Electronic Structure and Laser, FORTH, Heraklion 71110, Crete, Greece; ‡ Department of Materials Science and Technology, University of Crete, Heraklion 71003, Crete, Greece; § Faculty of Physics, University of Vienna, Boltzmanngasse 5, A-1090 Vienna, Austria; ∥ Physico-Chimie des électrolytes et Nanosystèmes Interfaciaux, Sorbonne Université CNRS, F-75005 Paris, France; ⊥ Polymer Synthesis Laboratory, Chemistry Program, KAUST Catalysis Center, Physical Sciences and Engineering Division, King Abdullah University of Science and Technology (KAUST), Thuwal 23955, Kingdom of Saudi Arabia; # Materials Science and Engineering, Grainger College of Engineering, University of Illinois, Urbana−Champaign, Illinois 61801, United States; ∇ Laboratoire Charles Coulomb (L2C), University of Montpellier, 34090 Montpellier, France; ○ Institut Universitaire de France, IUF, 75231 Paris, Cedex 05, France; ◆ Niels Bohr Institute, University of Copenhagen, Universitetsparken 5, 2100 Copenhagen Ø, Denmark; ¶ Department of Chemical and Biological Engineering, Princeton University, Princeton, New Jersey 08544, United States

## Abstract

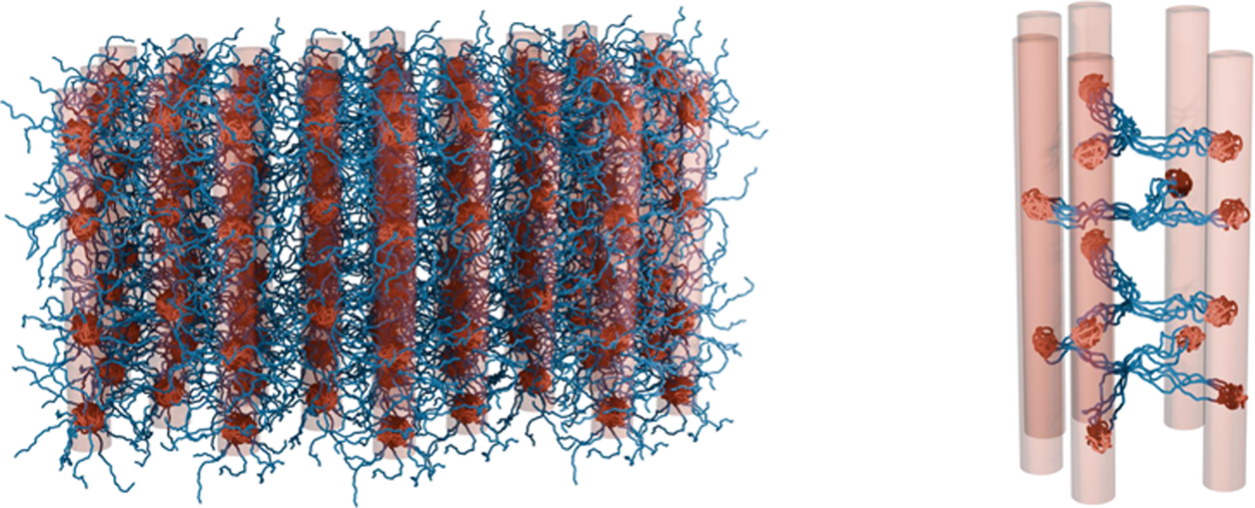

The design of functional polymeric materials with tunable response requires a synergetic use of macromolecular architecture and interactions. Here, we combine experiments with computer simulations to demonstrate how physical properties of gels can be tailored at the molecular level, using star block copolymers with alternating block sequences as a paradigm. Telechelic star polymers containing attractive outer blocks self-assemble into soft patchy nanoparticles, whereas their mirror-image inverted architecture with inner attractive blocks yields micelles. In concentrated solutions, bridged and interpenetrated hexagonally packed nanocylinders are formed, respectively, with distinct structural and rheological properties. The phase diagrams exhibit a peculiar re-entrance where the hexagonal phase melts upon both heating and cooling because of solvent–block and block–block interactions. The bridged nanostructure is characterized by similar deformability, extended structural coherence, enhanced elasticity, and yield stress compared to micelles or typical colloidal gels, which make them promising and versatile materials for diverse applications.

## I Introduction

Hierarchical self-assembly, the fundamental mechanism to form reversible multiscale material structures, is ubiquitous in modern technology since the properties of new products depend on the type of building blocks.^1^ Introducing directional interactions to building blocks via patchiness greatly enriches the realm of possibilities in material science.^1,2^ The anisotropic nature of patchy interactions limits the number of valences that each particle can have, which allows the formation of low-density gels,^3^ open crystals,^4,5^ and cluster phases.^6,7^ DNA-coated colloidal particles are one popular example of such soft patchy building blocks. In such suspensions, colloidal particles aggregate when the temperature is reduced below a certain system-dependent value as a result of interparticle binding.^8−14^ An appropriate deposition (or programming) of DNA-coated particles allows for the coupling of inter- and intraparticle binding.^15^ The fine-tuning of such competing interactions can lead to complex self-assembled structures with rich phase behavior characterized by colloidal crystallization^15^ or gelation^16,17^ on cooling, followed by re-entrant melting on further cooling. However, DNA technology is often complex, and among the many outstanding challenges for applications, combining simplicity, versatility, and responsiveness stands out.^18,19^ Moreover, introducing intraparticle associations requires complex grafting of DNA strands on binary mixtures of colloidal particles. A simpler way to introduce competitive interactions can be achieved by combining macromolecular architecture and enthalpic interactions and is implemented with a special class of star polymers, the telechelic star polymers (TSPs).^20−22^ TSPs are macromolecules made of *f* diblock copolymers grafted on a central anchoring point. Each diblock arm has a fraction α of solvophobic (at the outside) and 1 – α of solvophilic monomers. The dual nature of their arms makes TSPs particularly sensitive to variations of external conditions such as temperature or ionic strength, which allows each particle to self-assemble into a soft entity with attractive patches on its surface.^23,24^ Such star block copolymers are different from linear block copolymers and, in particular, more versatile. For example, a linear ABA triblock copolymer forms only one intramolecular loop or intermolecular bridge via its A blocks, whereas a TSP may form more loops or more bridges, as discussed below.^24−26^

A straightforward, robust method to tune the interparticle interactions in such a model system emerges: by changing the solvent quality in TSP solutions, it is possible to cover the entire range from purely repulsive (mutually good solvent) to attractive soft colloids, whose softness depends primarily on the star functionality *f*. The TSPs form patches when the temperature is reduced below the Θ-value of the outer block (the inner being under athermal conditions), which result from intra- and intermolecular attractions, depending on temperature, concentration, and fraction of the attractive outer block. TSPs with low functionality form micellar aggregates at low concentrations,^25−28^ which, at higher concentrations, lead to the formation of wormlike micelles.^25,29^ By contrast, TSPs with high functionality form ordered lattices having coordination compatible with the number of patches of a single TSP, making TSPs useful tunable building blocks for the formation of multiscale hierarchical supramolecular structures.^30^

Given the relative simplicity of the TSP building blocks, a formidable challenge and opportunity emerge to explore and exploit the properties stemming from the tunability of star block copolymers in different environments. In particular, the use of selective solvents for either block of the star block copolymer represents the design parameter. Here, we show how to take advantage of the duality of these materials by using a star block copolymer with inverted block sequences. We synthesized stars of intermediate functionality *f* = 16, where each arm is made of a diblock copolymer of polystyrene (PS) and polyisoprene (PI) with nearly the same molecular weight (see Section II for details). Two different sets of such stars were prepared, one having PS as the outer block and the other one having PS as the inner block. Below its cloud point, the PS block acts as the attractive element of the star for 22 °C < *T* < 53.5 °C. Hence, referring to a single-molecule structure, with the PS block outside, a TSP is formed, whereas the case of an attractive PS block at the star core corresponds to a micelle. By combining diverse experiments and coarse-grained simulations, we discover new ordering phenomena and demonstrate the possibility to tailor the behavior of these materials from predominantly disordered liquids to different types of crystals. The experimental observation of re-entrant melting with such simple and easy to handle star block copolymers, confirmed by simulations, is promising for exploring the rich physics of ordering transitions in mesophases. Our results reveal that ordered TSP solutions form interconnected (bridged) nanocylinders with a higher degree of structural coherence in comparison to the interpenetrated nanocylinders from the inverted star copolymer architecture, resulting in enhanced viscoelastic properties and yield stress. The proposed approach paves the way for making versatile networks with tunable properties, which make them promising for numerous applications ranging from membranes and films to drug delivery.

## II Materials and Methods

### II.I Synthesis and Characterization

Star block copolymers with *f* = 16 arms were used. Each arm was a diblock copolymer of polyisoprene (PI) and polystyrene (PS) with nearly identical weight-average molar mass *M*_W_ of about 26,000 g mol^–1^ (with a polydispersity of 1.12), hence, with a weight fraction of the attractive block of about 0.5. We used two such copolymers with different block sequences, one having PS inner block and another having PI inner block. These 16-arm star block copolymers were synthesized by combining anionic polymerization with chlorosilane linking chemistry using high-vacuum techniques. Living linear diblock copolymers were first synthesized by sequential block copolymerization of S(I) and I(S) in benzene with *sec*-butyllithium as initiator, followed by a reaction with a linking agent with 16 chlorosilane bonds (2G-Cl). The linking agent was synthesized by hydrosilylation of vinylsilane with dichloromethylsilane, followed by a reaction with vinylmagnesium bromide and another hydrosilylation with dichloromethylsilane. Details about synthesis and characterization are presented previously^31^ and in the Supporting Information (SI) (see Table S1 and Figures S18–S23). Note that we performed several fractionations to remove the precursors, but, unfortunately, it was impossible to completely remove them. We could perform more fractionations to completely remove the precursor at a cost that would not be sufficient to perform the array of experimental studies discussed below. Hence, despite the fact that these samples are the best possible 16-arm-star block copolymers, obtained solely through tedious high-vacuum techniques, there are tiny impurities discerned in the SEC traces; we shall comment on their possible effect below. The solvent of choice was 1-phenyldodecane, which, for dilute solutions, has a cloud point of about 53.5 °C for PS^32^ (also measured independently in a dilute solution of linear PS with *M*_W_ = 900,000 g mol^–1^) and 22 °C for PI (in dilute solution for linear PI with *M*_W_ = 300,000 g mol^–1^). The corresponding Θ temperatures are expected to be slightly higher. Given the very limited amounts of stars available, the choice of very high molar mass of linear polymers (compared to the stars) was dictated by the fact that the cloud point of linear polymers increases with the molar mass at upper critical solution temperature conditions, and at the same time, star polymers with slightly different near-core conformation exhibit a slightly higher cloud temperature compared to their linear counterparts. Here, we use the so-determined cloud points as a guide, determine the transitions based on the distinct rheological and scattering features, and construct the phase diagram based on them, as discussed below. In addition, the chosen solvent has a boiling point of 330 °C at atmospheric pressure; hence, it is amenable to long-time rheological experiments. In the range 22 °C < *T* < 53.5 °C, the PS block acts as the attractive element of the star. Hence, when the PS block is outside, we have a telechelic star polymer (TSP) or a patchy particle, whereas when the attractive PS block is inside, we have a micelle-like architecture. Therefore, the former system is referred to as TSP and the latter as micelle.

Solutions were prepared by mixing an appropriate amount of the star block copolymer with the solvent to reach the desired concentration. The sample stability was ensured by adding 0.1% w/w of antioxidant BHT (2,6-di*tert*-butyl-4-methylphenol). In order to fully dissolve TSPs, methylene chloride was used as the cosolvent and subsequently evaporated under ambient conditions until a constant weight was achieved. The dilute solution characterization with dynamic light scattering (DLS) suggests that intra- and interstar association of PS blocks take place when the temperature is reduced below the cloud point of the outer PS block, the former prevailing at a larger fraction of PS (as in the present case of 0.5) and the latter at a lower fraction of PS (see Figures S1 and S2). This picture conforms to recent simulation results^24,33^ and DLS characterization on similar TSPs in selective solvents.^33,34^

### II.II Experimental Techniques

Rheological experiments were performed on an ARES-HR (TA) sensitive strain-controlled rheometer with a stainless steel cone and plate geometry (diameter 8 mm, angle 0.166 rad, and truncation 0.21 mm). Temperature control in the range of 10 to 100 °C (±0.1 °C) was achieved by means of a Peltier element. In order to erase thermal history effects, before each experiment, the sample was heated well above the order–disorder transition temperature and kept for about 10 min, which was found to be sufficient for the system to reach equilibrium, as inferred from the time-independent linear viscoelastic moduli. The system was then set to the desired temperature and let to rest toward equilibrium for a time that depended on temperature. Typically, at temperatures above but near the order-to-disorder transition, the rest time was up to 20 min. At and below the transition, the sample would age for a time between 1 and 5 h, as judged by nearly time-independent moduli. For some TSPs at the lowest temperatures, we used longer rest times (24 h) and did not observe appreciable differences. Finally, small amplitude oscillatory shear (hereafter called dynamic frequency sweep (DFS)) measurements were performed in the linear viscoelastic regime with a strain amplitude of 1% over a frequency range of 100–0.01 rad s^–1^. The limit of the linear viscoelastic regime was determined by performing dynamic strain sweep experiments at various frequencies. Although the used protocol leads to reproducible results, we did not investigate the kinetics of phase transitions, and therefore the possible effects on the transitions and/or crystallinity are not considered. Nonlinear measurements (start-up in shear) are discussed in Section III.V below.

Small-angle X-ray scattering (SAXS) experiments were performed using the GANESHA-SAXS/WAXS instrument from SAXSLAB installed at the Niels Bohr Institute, University of Copenhagen. The instrument is equipped with a GeniX-3D microfocus sealed X-ray tube (Xenocs, France), a multilayer focusing mirror system, and a two-dimensional 300k Pilatus detector from Dectris (Switzerland). Measurements were performed with a “two pinhole” collimated beam using scatterless slits and a 1 m sample-to-detector distance. With this setup, we obtained a *q*-range from 0.005 to 0.28 Å^–1^. Here, the protocol was slightly different. Starting from the homogeneous regime, the temperature was gradually reduced to the set value, and rest times similar to rheology were used. However, for TSPs at the lowest temperatures, the rest time was only 3 h. Hence, some differences in the ordered structures and transitions cannot be excluded. We did not attempt to quantify the crystallinity.

Multispeckle dynamic light scattering (MDLS) measurements were run on the setup described in the literature.^35,36^ In brief, a laser beam with in-vacuum wavelength λ = 532.5 nm, power 150 mW, and 1/*e*^2^ diameter = 1 mm illuminated the sample, contained in a NMR tube placed in a temperature-controlled copper holder, which ensured temperature stability better than 0.1 °C over several hours. Light scattered at a scattering angle θ = 90° formed a speckle pattern, which was collected by a CMOS camera. A time series of speckle images was recorded using the variable delay time scheme of ref (37), allowing for spanning time delays τ between pairs of images from 10^–2^ to 10^4^ s. The speckle images were processed as described by Duri et al.^38^ to calculate the two-time degree of correlation , with *I*_*p*_ the time-dependent intensity of the *p*-th pixel and ⟨···⟩_*p*_ an average over pixels. The intensity autocorrelation function *g*_2_(τ) – 1 was obtained by averaging *c*_*I*_ (*t*, τ) over time *t*, ranged from a few hundred seconds to several hours, depending on the sample. As in conventional dynamic light scattering, *g*_2_(τ) – 1 is the square of the (coherent) intermediate scattering function, which quantifies the temporal relaxation of density fluctuations of wave vector , with *n* = 1.482 the index of refraction of the solvent and *q* the scattering vector and θ the scattering angle. Samples were fluidized by heating them at *T* = 70 °C for 30 min prior to measurements to erase any previous thermal history. They were then placed in the sample holder at the set temperature, and after temperature equilibration (with rest times similar to those in rheology), they were measured at time *t* = 0.

### II.III Simulation Methods

Star block copolymers in solution were simulated using dissipative particle dynamics (DPD)^39^ with explicit solvent particles to account for solvent selectivity and hydrodynamic effects. In short, the total force *F*_*i*_ acting of the *i*th particle in DPD is given by , where **F**_*ij*_^C^ = *A*_*ij*_*w*(*r̂*_*ij*_) is the conservative force between the *ij*-pair of particles at distance *r*_*ij*_ = |**r**_*ij*_| (note that here, and in what follows, ), **F**_*ij*_^D^ = −γ*w*^2^(*r̂*_*ij*_)(**r̂**_*ij*_·**v**_*ij*_) is the pairwise dissipative force, and  is the pairwise random force contribution, with η_*ij*_ being a Gaussian random number with zero mean and unit variance. Above, the function *w*(*r*_*ij*_) is given by , where θ(*x*) is the Heaviside step function and σ is the cutoff distance that sets the range of pairwise interactions and is also chosen as the unit of length in DPD (σ = 1). The parameter *A*_*ij*_ defines the strength of interparticle repulsion. The mass of all particles in the simulations *m* was chosen to be the same and thus served as the unit of mass (*m* = 1). *k*_B_*T* was chosen as the unit of energy (*k*_B_*T* = 1). The systems were simulated at particle density ρσ^3^ = 3 using γ = 4.5 *m*τ^–1^ and the Velocity-Verlet integration time step Δ*t* = 0.04τ, where  is the DPD unit of time. All simulations were performed using the HOOMD-blue simulation package on graphics processing units (GPUs).^40^

The DPD repulsion amplitudes *A*_*ij*_ are directly related to the Flory–Huggins parameters χ_*ij*_ that are inversely proportional to temperature and affect the phase behavior in polymer solutions^41^
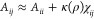
1where *A*_*ii*_ = 25 and κ(ρ) = 3.497 for the given density ρσ^3^ = 3.^39^ For the systems at hand, it is necessary to specify three such values: χ_AB_, χ_AS_, and χ_BS_. Here, A and B stand for the two monomer types, isoprene and styrene, respectively, and S denotes solvent particles. Given that  for PI–PS block copolymers,^42^ the values of χ_AB_*N* (*N* is the polymerization degree of a star arm) are 50 < χ_AB_*N* < 40 within the range of temperatures 10 °C < *T* < 60 °C relevant for this work. Such values of χ_AB_*N* are well into the ordered part of the phase diagram for star block copolymers of similar composition.^43^ Thus, in what follows, we assume that the phase behavior here is mainly controlled by the effect of solvent selectivity that arises from increasing values of χ_AS_ and χ_BS_ but not from the changes in χ_AB_. In practice, we fixed the value of χ_AB_*N* at an intermediate experimental value of (χ_AB_*N*)_exp_ = 45 and systematically varied χ_AS_ and χ_BS_.

We considered two star polymer models to assess the assembly of single stars in solution and their phase behavior in the concentrated regime. In both cases, stars with *f* = 16 arms contained *N*_A_ = *N*_B_ = *N*/2 monomers of each type that corresponds to α = 0.5. For single star modeling, the star arm length *N* was chosen to be *N* = 64. On the other hand, due to economy in modeling, in concentrated systems we used stars with shorter arms of length *N* = 10. Due to a small polymer chain length in the simulations, it is necessary to take into account finite polymer length corrections that result in an effective χ_AB_-parameter
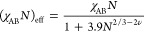
2where we used ν = 0.588 for the two cases here.^41^ Thus, χ_AB_ in DPD was chosen to match (χ_AB_*N*)_eff_ that corresponds to the experimental value of (χ_AB_*N*)_exp_ = 45. This results in χ_AB_ = 1.03 (*A*_AB_ = 28.6) for *N* = 64 and χ_AB_ = 9.92 (*A*_AB_ = 59.7) for *N* = 10. The values of χ_AS_ and χ_BS_ were systematically varied in both cases.

Single stars (*f* = 16, *N* = 64) for the telechelic, (A–B)_16_, and micellar, (B–A)_16_, architectures were simulated in a cubic box with side length *L* = 30σ for different values of χ_AS_ and χ_BS_. The polymer size as a function of the χ-parameters was assessed in terms of the star’s radius of gyration
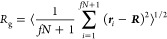
3where ***r***_*i*_ is the position of *i*th monomer, ***R*** is the position of star’s center of mass, and angles ⟨···⟩ stand for ensemble averaging. The star dynamics was quantified in terms of their center-of-mass mean square displacements (MSD)
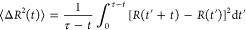
4with τ denoting here the total simulation time. The star’s center-of-mass diffusion coefficient *D* was extracted from the long-time behavior of the corresponding MSD
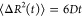
5

The static and dynamic properties of single stars were averaged over 6–12 independent simulation runs of 2.5 × 10^6^ DPD time steps. The resulting values of the diffusion coefficient were corrected for finite-size effects using the Yeh–Hummer formula^44^
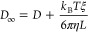
6where *D*_∞_ corresponds to a macroscopic system and *D* was obtained in a cubic box of side length *L* with periodic boundary conditions and ξ ≈ 2.837297. We used the solvent viscosity η for the given simulation parameters to estimate the finite-size correction.^45^

Concentrated star solutions (*f* = 16, *N* = 10) for the telechelic, (A–B)_16_, and micellar, (B–A)_16_, systems were simulated in a cubic box at different monomer volume fractions ϕ_p_ = 0.3–1.0, where ϕ_p_ = *N*_p_/(*N*_p_ + *N*_s_), with *N*_p_ being the total number monomers and *N*_s_ being the total number of solvent particles, and varying the values of χ_AS_ and χ_BS_ (since all particle sizes are the same, the volume fractions are the same as the corresponding number fractions). In each case, the system contained *M* = 500 polymer stars. For a given ϕ_p_, the solutions were equilibrated by gradually increasing all χ-parameters to the target values over 1.5 × 10^7^ DPD time steps. 1–3 independent simulation runs were performed to assess the final assembled state of the system.

To analyze the interconnectivity of the star network in the hexagonally ordered phase, we first identified formed cylinders in the main simulation box using the DBSCAN clustering algorithm.^61^ Subsequently, we determined the number of distinct cylinders that a given star belongs to for a series of equilibrated system snapshots with well-formed ordered phases. In practice, to avoid overcounting due to periodic boundary conditions, in the analysis, we only included those stars whose centers were located at a distance 1.5*R*_g_ away from the periodic boundaries.

## III Results and Discussion

### III.I Responsiveness of Star Block Copolymers: Self-Assembly at the Single-Molecule Level

**Figure 1:**
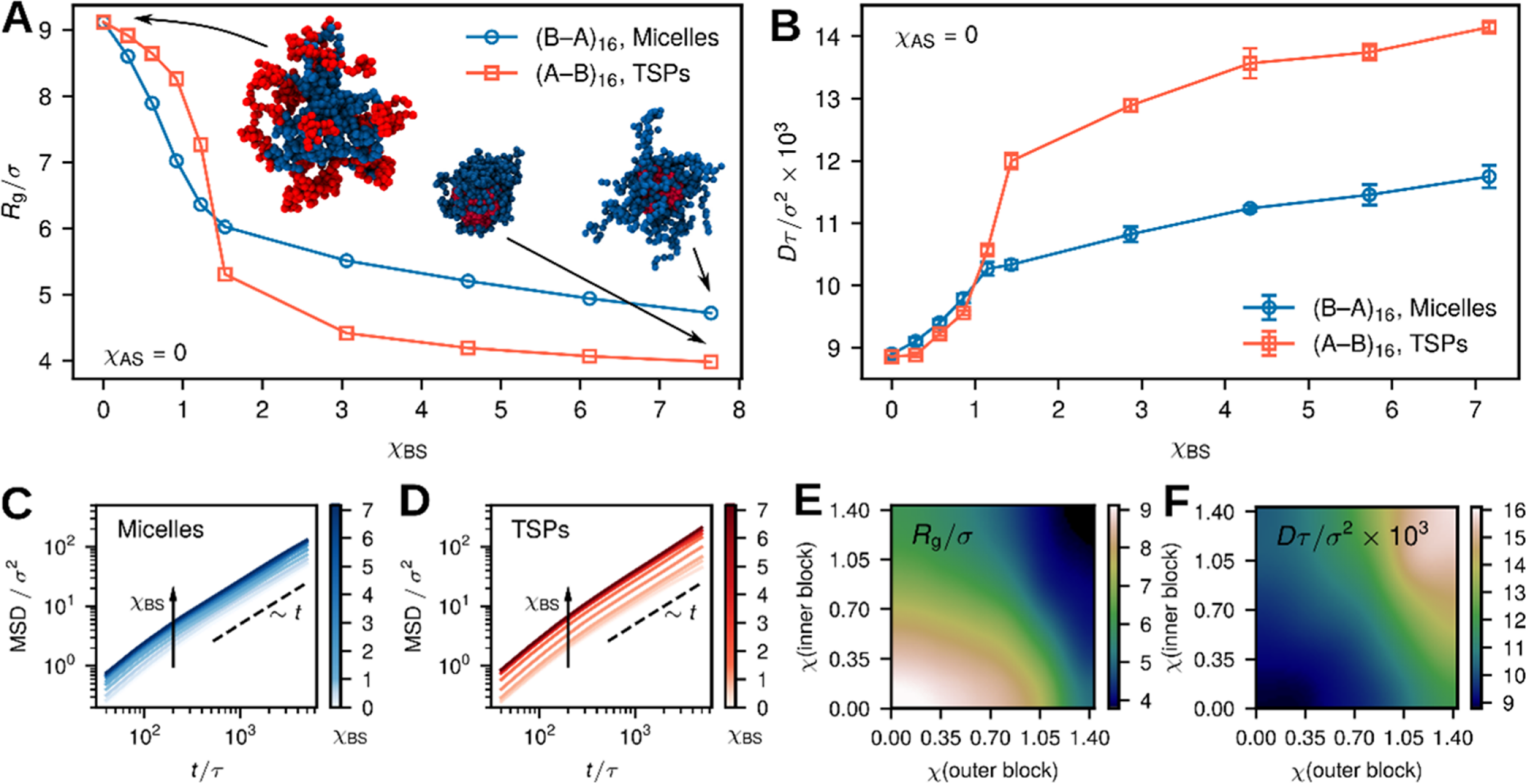
Conformations and dynamics of single stars. (A) Radius of gyration *R*_g_ of a single star as a function of the incompatibility parameter χ_BS_ between the solvent and B monomers for the micellar, (B–A)_16_ (blue open circles), and telechelic, (A–B)_16_ (red open squares), architectures. The snapshots in panel (A) indicate typical star conformations with red monomers corresponding to B blocks and blue monomers to A blocks: a telechelic (A–B)_16_ star polymer (TSP) in good solvent conditions (left), a collapsed TSP (center), and a collapsed (B–A)_16_ micelle (right). (B) Corresponding center-of-mass diffusion coefficient *D* for varying χ_BS_, as extracted from the mean square displacements of the star’s center of mass for the micellar (C) and telechelic (D) architectures. The A monomers in panels (A–D) are in good solvent conditions with χ_AS_ = 0. A generic two-dimensional phase diagram for single star’s *R*_g_ (E) and *D* (F) in the case of varying incompatibility parameters between the solvent and inner, χ(inner block), and outer, χ(outer block), blocks. In all simulations here, we used the value χ_AB_ = 1.03, which corresponds to the experimental PS–PI copolymer within the relevant range of temperatures (see Section II). The values of the diffusion coefficient were corrected for finite-size effects (see Section II).

To appreciate the ability of the star block copolymers to attain a variety of conformations in different environments, we start with exploring the self-assembly patterns of a single such molecule in selective solvents using coarse-grained computer simulations, as highlighted in Figure 1. We focus on mimicking the two experimental systems: TSPs (having attractive B-block outside), (A–B)_16_, and micelles (attractive B-block inside), (B–A)_16_, with equal lengths of two blocks, α = 0.5. Here, the stars with *f* = 16 arms of length *N* = 64 were simulated using dissipative particle dynamics (DPD) with explicit solvent particles and varying solvent quality, as quantified by means of the Flory–Huggins parameters χ_BS_, χ_AS_, and χ_AB_ (see Section II for details). Finally, note that the model employed is not a result of systematic coarse-graining that would retain the details of microscopic interactions necessary for a quantitative comparison against the experiments. Rather, it aims at rationalizing generic trends in system assembly as a function of the main control parameters.^23,30,31,33^

Figure 1A shows the size of a single star, quantified in terms of its radius of gyration *R*_g_ (eq 3 in Section II), as a function of the increasing Flory–Huggins parameter χ_BS_ between the B-blocks and the solvent particles for χ_AS_ = 0 (good solvent conditions) and χ_AB_ = 1.03 (the latter value was chosen to resemble the experimental PS–PI copolymer, as we show in Section II). Therefore, such simulations correspond to A blocks in good solvent and B blocks in worsening solvent conditions, with A and B standing for PI and PS here, respectively. This effectively models the experimental PS–PI system upon decreasing the temperature below the Θ-value of PS, *T*_Θ_^PS^, but keeping it above *T*_Θ_^PI^. For χ_BS_ = 0, the two polymer architectures are subject to good solvent conditions with the same *R*_g_ (a typical TSP conformation is shown in the top leftmost inset of Figure 1A with red B and blue A monomers). For both systems, we find a sharp decrease in the simulated star’s *R*_g_ for χ_BS_ ≳ 1.4 associated with the formation of a single, bulky patch between outer B blocks for the TSP and the collapse of inner B blocks for the micelles (see the central and rightmost insets of Figure 1A, respectively).^22−29^ Dynamic light scattering experiments in the dilute solution also exhibit a similar reduction of the hydrodynamic size on worsening solvent quality for the PS block (see Figures S1–S3 for the present TSPs with *f* = 16 and also the recent literature^31,33^ for TSPs with a lower functionality of *f* = 3). The significant change occurs at the same temperature (Θ-point of PS block) for both micelles and TSPs. Note, however, that the comparison between simulations and experiments for both *f* = 16 and 3 is qualitative, since the former attempts to mimic but not replicate the real system by employing a generic form of the interactions between monomers and solvent particles (see also Section II and the Supporting Information). Furthermore, due to architectural differences, the TSP features more compact conformations compared to the micelles in the assembled state at large values of χ_BS_ (Figure 1A) because, in TSPs, the outer cores collapse, while in micelles, the inner cores do and the outer cores remain hairy. It should be noted that a detailed match between experiments and simulations requires accurately matched microscopic models at different scales. While such models may be formulated at the atomistic level, the development of DPD models requires reparametrization of the force fields and overcoming computational restrictions, a challenging task that goes beyond the scope of the present work. Thus, we simulate here the systems at the very coarse level and take into account only the effective Flory parameters, which we consider to be the most important set of parameters to tune the phase separation between species.

The drop in the star size is accompanied by a marked increase in the stars’ diffusion coefficient *D* (Figure 1B), which we extract from the long-time behavior of their center-of-mass mean square displacements (MSD) shown in Figure 1C,D (see eqs 4–6 in Section II). At χ_BS_ = 1.4, when both star architectures feature an assembled state, the diffusion coefficient of the TSP is about 50% higher than that of the micelle, while its *R*_g_ is only about 15% smaller. The latter is caused by the fact that the collapsed (B–A)_16_ stars feature a “hairy” shape (the rightmost snapshot of Figure 1A) with A blocks frequently interacting with the solvent and thus decreasing the polymer’s diffusion. Upon further increasing χ_BS_ for both architectures, their *R*_g_ slowly decreases and *D* increases, respectively (note, however, that at this level of coarse-grained modeling, there is no evidence of significant rearrangement in the microstructural rearrangements of the star polymers). To assess the effect of solvent quality with respect to A blocks (corresponding to experimentally decreasing temperature beyond *T*_Θ_^PI^), in Figure 1E,F, we show a generic dependence of *R*_g_ and *D* on the incompatibility parameter between the solvent and inner, χ(inner block), and outer, χ(outer block) blocks. A clear anticorrelation between *R*_g_ and *D* is evident. Interestingly, due to incompatibility between A and B monomers, when both blocks are in poor solvent conditions (χ_AS_, χ_BS_ ≳ 1), the star assembles into a large, very compact Janus-like particle (see snapshots in Figure 1A)^6,23^ with a diffusion coefficient that exceeds that of the TSP and the micelle having only the B block in a poor solvent (Figure 1E,F).

### III.II Self-Assembly and Dynamics in the Concentrated Regime

**Figure 2:**
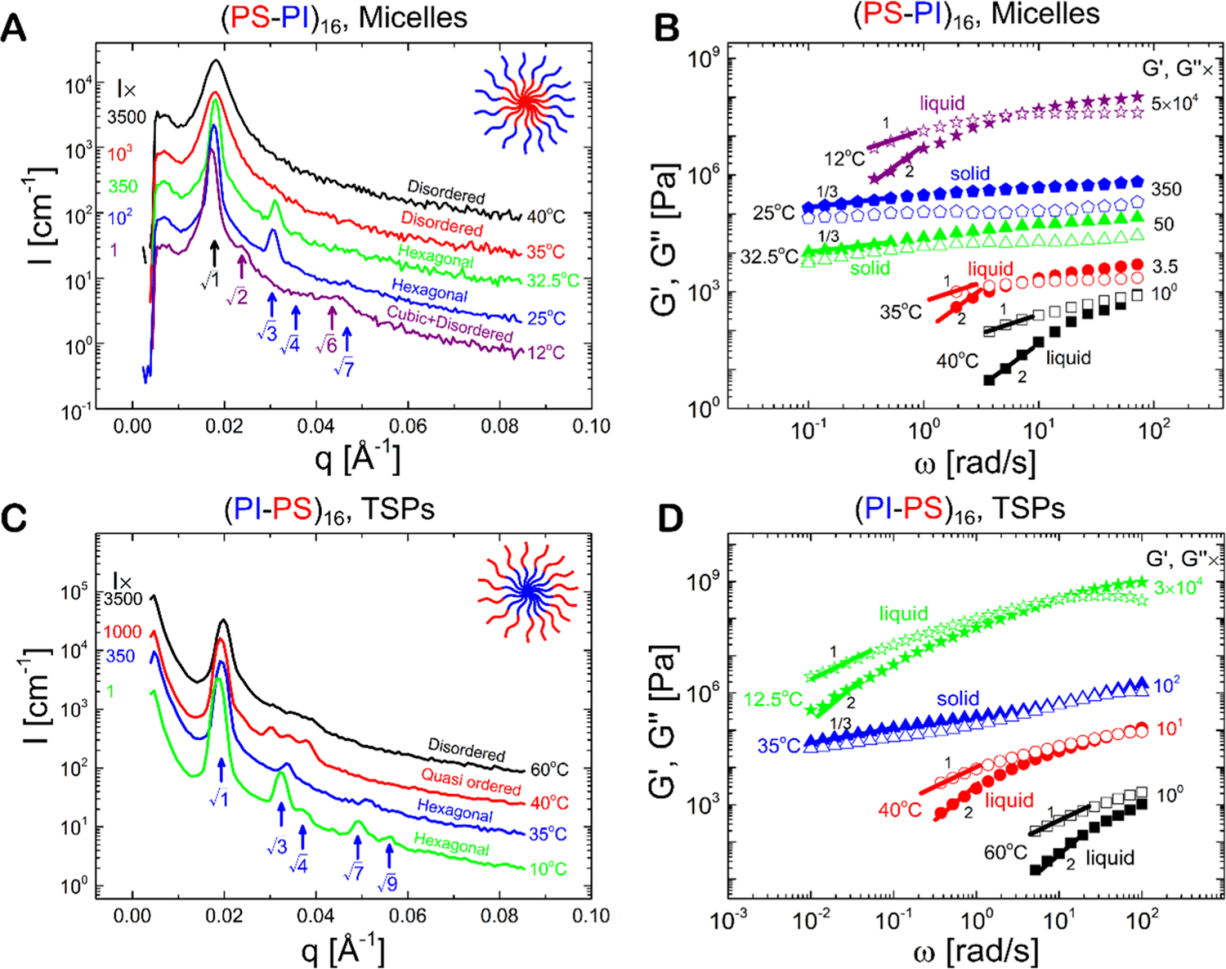
Structural and rheological properties of star block copolymers in the concentrated regime. (A) Representative SAXS profiles and (B) dynamic frequency spectra depicting the storage *G*′ (closed symbols) and loss *G*″ (open symbols) moduli as a function of angular frequency at different temperatures (marked in the legend) for the star block copolymer with PS as the inner block (micelles) and the star mass concentration of 27% w/w. Panels (C, D) are as panels (A, B), respectively, but for the star block copolymer with PS as the outer block (TSPs) and a star mass concentration of 33% w/w. The blue-colored numbers in panels (A, C) refer to the relative positions for the first few reflections for the hexagonal order, and the purple-colored numbers in panel (A) represent the reflections for the cubic order. The solid lines in panels (B, D) show the low-frequency power-law slope of the moduli. For clarity, values of intensity and viscoelastic moduli are shifted vertically, as shown in the legend.

For each star block copolymer system, TSPs (PS at the outer block), and micelles (PI at the outer block), we investigated the variation of structure and dynamics in the concentration–temperature phase space by means of small-angle X-ray scattering (SAXS), linear viscoelastic (dynamic frequency sweep, DFS), and light scattering (multispeckle dynamic light scattering, MDLS) measurements (see Section II for details). SAXS and rheological results for the micellar system at a concentration of 27% w/w and varying temperatures are presented in Figure 2A,B, which depicts the *q*-dependent SAXS intensity and viscoelastic spectra, respectively. At the higher temperature of 40 °C, a well-defined broad peak appears in the SAXS data at *q* = 0.018 Å^–1^, corresponding to a length scale of 345 Å (nearly 2 times the hydrodynamic radius, 2*R*_h_) without evidence of long-range order. Along with the respective rheological data (with larger values of loss modulus *G*″ compared to the storage modulus *G*′ and terminal flow scaling, *G*′ ∼ ω^2^ < *G*″ ∼ ω, at the accessible lowest frequencies, see Figure 2B) and the evolution of the data at lower temperatures, this strongly suggests that the solution is a disordered viscoelastic liquid. When the temperature is reduced to 35 °C, the structure remains disordered, and the solution still exhibits a liquid-like response, however, with much slower dynamics (even when accounting for the increase of solvent viscosity), as shown in Figure 2B by the shift of the terminal crossover frequency to a lower value. A further small decrease of temperature to 32.5 °C leads to a drastic change in both structure and dynamics. The first-order peak in SAXS sharpens dramatically (see also Figure S4), and an additional peak emerges at a *q*-value corresponding to √3-times the first-order peak. These findings indicate that the stars become well-positioned in space; hence, a disorder-to-order transition takes place upon cooling. Concomitantly, the solution exhibits a transition from liquid-like to solid-like response marked by *G*′ > *G*″ over the entire range of examined frequencies. With a subsequent decrease of the temperature, the higher-order peaks are clearly evidenced and become stronger, reflecting a more coherent organization. Analysis of the SAXS data yields the assignment of the intensity peaks to hexagonally packed cylindrical structures (in this regard, the dominant first order and √3-peaks are important). This is corroborated by the low-frequency moduli, which follow a power-law scaling *G*′ ∼ *G*″ ∼ ω^α^ with , i.e., the rheological fingerprint for hexagonally packed cylinders.^46,47^ Of course, this scaling is reached at the lowest frequencies (a range less than a decade), so we refrain from speculating on a possibly extended power-law behavior expected for well-discerned hexagonal phases; nevertheless, this observation is quite revealing. When the temperature is decreased below the cloud temperature of the outer PI block (22 °C), a weak order-to-order transition (OOT) is detected. The SAXS pattern with peaks 1:√2:√6 indicates cubic order. The intensities of the higher-order peaks are relatively weak, and the first-order peak becomes slightly broader (see Figure S4A), suggesting the absence of true long-range order, in agreement with the rheological data, which point to a re-entrant liquid-like response (Figure 2B). Hence, the emerging picture is that of an incomplete cubic order with a significantly disordered structure or a mix of melted and cubic phases. Such defect-mediated cubic structure is expected to exhibit a predominantly liquid-like response arising from the relaxation of mobile defects^48,49^ (for example, the impurities mentioned above).

Next, we discuss the respective structural and rheological properties of a TSP at a concentration of 33% w/w (Figure 2C,D). At the highest temperature of 60 °C, the structure is essentially disordered but with some very weak higher-order correlation peaks. The rheology detects a well-defined liquid-like response. Upon cooling to 40 °C, the SAXS data indicate a transition to an ordered structure. One may speculate a texture with a double diamond arrangement (see Figure S5 for structural analysis). Other possibilities are that the structure is (slightly) noncubic or a mixture of more (mostly cubic) phases. We refer to this rearrangement as quasi-ordered structure, since the TSP with well-defined (though weak) Bragg peaks exhibits the rheological response of a viscoelastic liquid. Indeed, at 40 °C, rheology still identifies a liquid-like response, however, with a distinct terminal dynamics, which is characterized by very broad relaxation of the moduli that are collapsed at higher frequencies and eventually reach the slopes of 1 and 2. This does not seem to be the case for the disordered liquid detected at the high temperature of 60 °C, in the accessible frequency range, and the broad terminal relaxation is clearly evident in the van Gurp–Palmen representation of the viscoelastic data, where a shoulder is discerned at intermediate values of complex modulus for 40 °C ≤ *T* ≤ 37.5 °C (Figure S6). When the temperature is further decreased to 35 °C, the SAXS data indicate that an OOT takes place from the quasi-ordered structure to a hexagonal arrangement. The latter is supported by the respective rheological signature of a hexagonal ordered solid, as in the micellar case.^46,47^ Further reduction of the temperature to 12.5 °C (well below the cloud point of the inner PI block, 22 °C) yields rheological re-entrant melting, while the structure remains hexagonal according to SAXS data at 10 °C. However, a careful inspection of the first-order peak indicates a continuous growth of the peak broadness upon reducing *T* below 20 °C (see Figure S7). This strongly suggests that the structure becomes less coherent on cooling below 20 °C, which eventually leads to a liquid-like character at lower temperatures. The changes in structure and dynamics at various temperatures for concentrations of 30% w/w and 40% w/w are shown in Figures S8–S12, respectively.

The central message from the rheological and SAXS studies is the unambiguous tunability of the dynamic response of micelles and TSPs as the temperature is reduced from the amorphous liquid regime. However, the formed ordered phases in the intermediate-temperature and the low-temperature re-entrant regimes are not as coherent and unambiguous as in other block copolymer cylindrical phases.^33^

**Figure 3:**
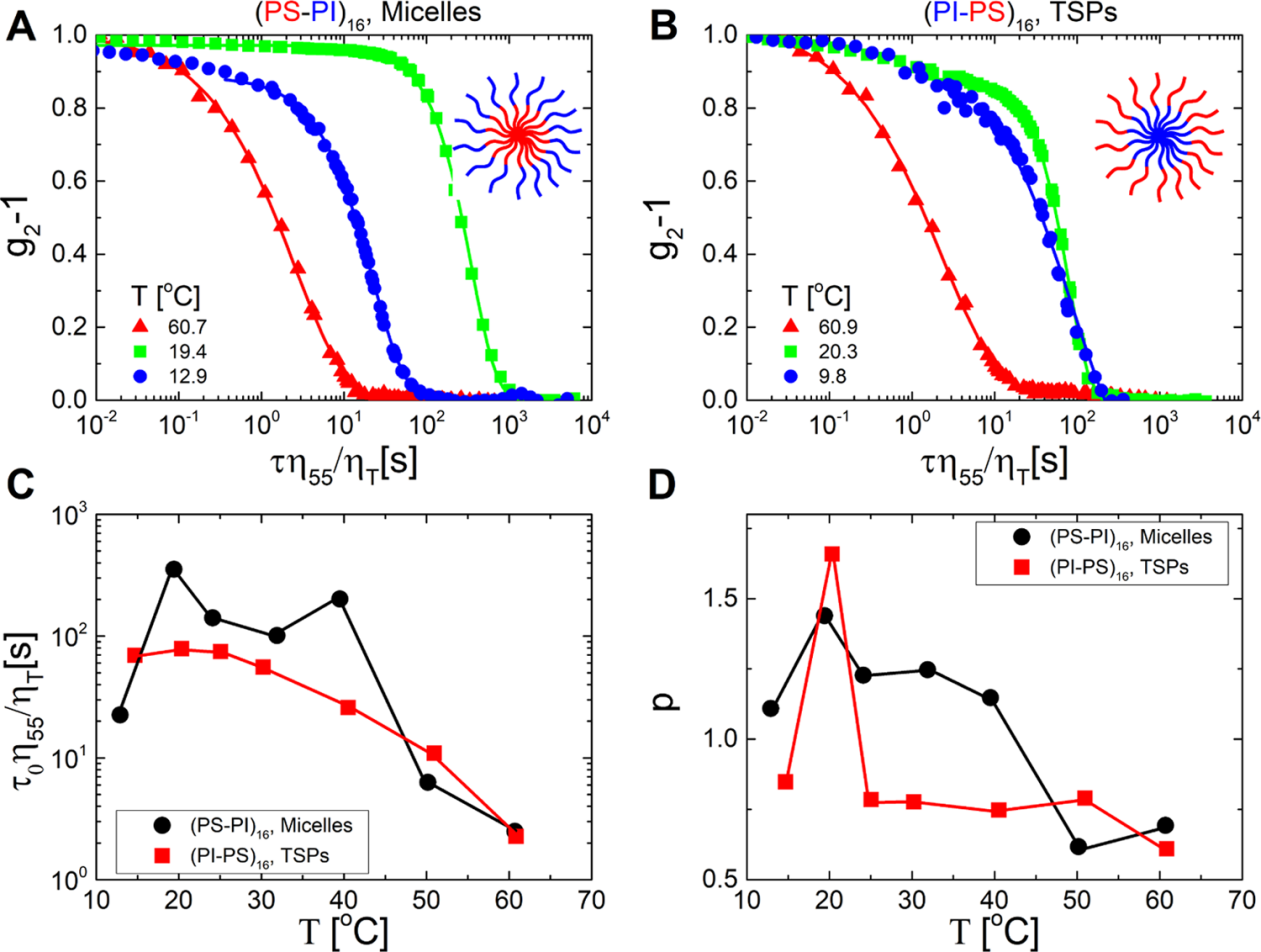
Microscopic dynamics of star block copolymers in the concentrated regime. Representative intensity correlation functions *g*_2_ – 1 measured at a wave vector *q* = 24.7 μm^–1^ at different temperatures (shown in the legend) for the micellar (A) and TSPs (B) systems. The solid lines in panels (A, B) represent stretched exponential function fits. (C) Temperature dependence of the relaxation time of *g*_2_ – 1 as obtained from stretched exponential fits. (D) Same as in (C), but for the stretching exponent *p*. In both systems, the star concentration is 30% w/w. In panels (A–C), time delays have been normalized to account for the change of solvent viscosity, η_T_, with temperature. The solvent viscosity at *T* = 55 °C, η_55_, is taken as a reference value (it corresponds to a homogeneous regime, above the cloud point of the PS block). The temperature dependence of solvent viscosity is shown in Figure S13. In panels (C, D), the error bars as determined from the fitting routine are not plotted, as they are smaller than or comparable to the symbol size: the percentage errors on time and *p* (panels C, D, respectively) are smaller than 5.6 and 3.6%.

To elucidate the microscopic dynamics of the micelles and TSPs across the various phases identified by SAXS and rheology, we performed MDLS measurements. We used a setup that allows probing the microscopic dynamics on a typical length scale of the order of *q*^–1^ = 40 nm and time scales from 10 ms to several hours (see Section II for details). Figure 3A,B shows representative intensity correlation functions (ICFs) at a fixed value of the scattering wave vector *q* = 24.7 μm^–1^ for both micelles and TSPs, respectively, at a concentration of 30% w/w and three temperatures. Time delays have been normalized to account for the change of solvent viscosity with *T*. In both systems, a decrease in temperature from ∼60 to ∼20 °C dramatically slows down the ICF, concurring with the rheological findings. Note that, despite the viscoelastic solid-like character at *T* = 20 °C, the ICF fully decays on time scales from hundreds to thousands of seconds. This indicates that density fluctuations relax, albeit very slowly, at the short length scales probed by MDLS. In contrast, at the macroscopic scales probed by rheology, a complete stress relaxation would require time scales longer than those (about 10^2^ s) accessed experimentally by rheology. This result is reminiscent of findings for other soft solids, e.g., colloidal gels,^50,51^ which exhibit solid-like rheological behavior concomitantly to ultraslow full relaxations of the ICF. A further reduction of temperature below *T* = 20 °C speeds up the dynamics, resulting in a nonmonotonic temperature dependence of the microscopic dynamics. This re-entrant effect is similar to that observed by rheology. In MDLS, re-entrance is more pronounced for the micellar system. For TSPs, the ICFs at *T* = 20.3 and 9.8 °C exhibit a two-step decay, indicative of a more complex nature of relaxation in TSPs as compared to micelles, which is again consistent with the rheological results.

In order to quantify the characteristic time scale of the decorrelation process, the final decay of the correlation function was fitted with a stretched exponential function, *g*_2_(τ) – 1 ∼ exp(−*t*/τ_0_)^*p*^. Figure 3C depicts the relaxation time τ_0_ extracted from the fit (and scaled appropriately to account for changes in the solvent viscosity) as a function of temperature. For both systems, the relaxation time increases by more than 1 order of magnitude on cooling to *T* ≈ 20 °C, while it decreases for *T* < 20 °C, i.e., below the cloud point of the PI block, in support of the re-entrant behavior suggested by the representative ICFs shown in Figure 3A,B and the rheological data of Figure 2B,D. The acceleration of the dynamics below 20 °C is more pronounced for the micelles than for TSPs. This observed re-entrant response differs from the rheological data, which showed a significant effect for both systems and was even more pronounced for the TSPs. We may attribute this to two possible reasons: the effect of impurities mentioned above and the associated different sensitivities of the two techniques in detecting the dynamic response. Indeed, MDLS is sensitive to the dynamics on local length scales (a few tens of nm), over which even macroscopically solid-like systems may exhibit relaxations on time scales only marginally slower than in viscoelastic fluids, due to the relaxation of internal stress, as briefly discussed below. Finally, we also note that the protocol used in the two techniques was similar but not identical.

Figure 3D depicts the temperature dependence of the stretching exponent *p*. A value of *p* < 1 indicates a stretched exponential relaxation, which is typical of dense fluids. By contrast, *p* > 1 represents compressed exponential relaxations, which are typically observed in viscoelastic solids having frozen-in stresses that are responsible for nearly ballistic dynamics.^35,51−54^ For the micellar system, *p* increases on cooling from 60.7 to 19.4 °C, starting with a value slightly below 1 at 60.7 °C and reaching a maximum of about 1.5 at 19.4 °C. This signals the response of a viscoelastic solid, in agreement with the rheological findings. Furthermore, by inspecting the two-time degree of correlation *c*_*I*_ (see Section II), we find that for *T* > 20 °C, the dynamics are stationary, as expected for a fluid. However, at *T* = 19.4 °C, the micellar system exhibits a slow albeit steady increase of the relaxation time with age during over 36 h, before reaching a nearly stationary state in the following 5 h, over which we average the ICF presented in Figure 3A. The lack of an unambiguously stationary state at *T* = 19.4 °C is again consistent with the picture of a (not fully equilibrated) viscoelastic solid, complying with the rheological results. Further cooling reduces the *p* value, suggesting that internal stresses are weakened, consistent with the hypothesis of partial melting of the sample. On the other hand, TSPs have *p* values nearly equal to 1 throughout the range 20 °C < *T* < 60 °C. Interestingly, at *T* = 20 °C, *p* significantly increases to 1.6, a value indicative of (nonequilibrated) solid-like behavior. For *T* < 20 °C, *p* drops to a value close to 1, reflecting relaxation of internal stresses and melting of the viscoelastic solid, similarly to the micellar system. To summarize, MDLS experiments confirm at the microscopic level the re-entrant scenario inferred from the rheological measurements and provide insights into its origin.

### III.III Phase Behavior

**Figure 4:**
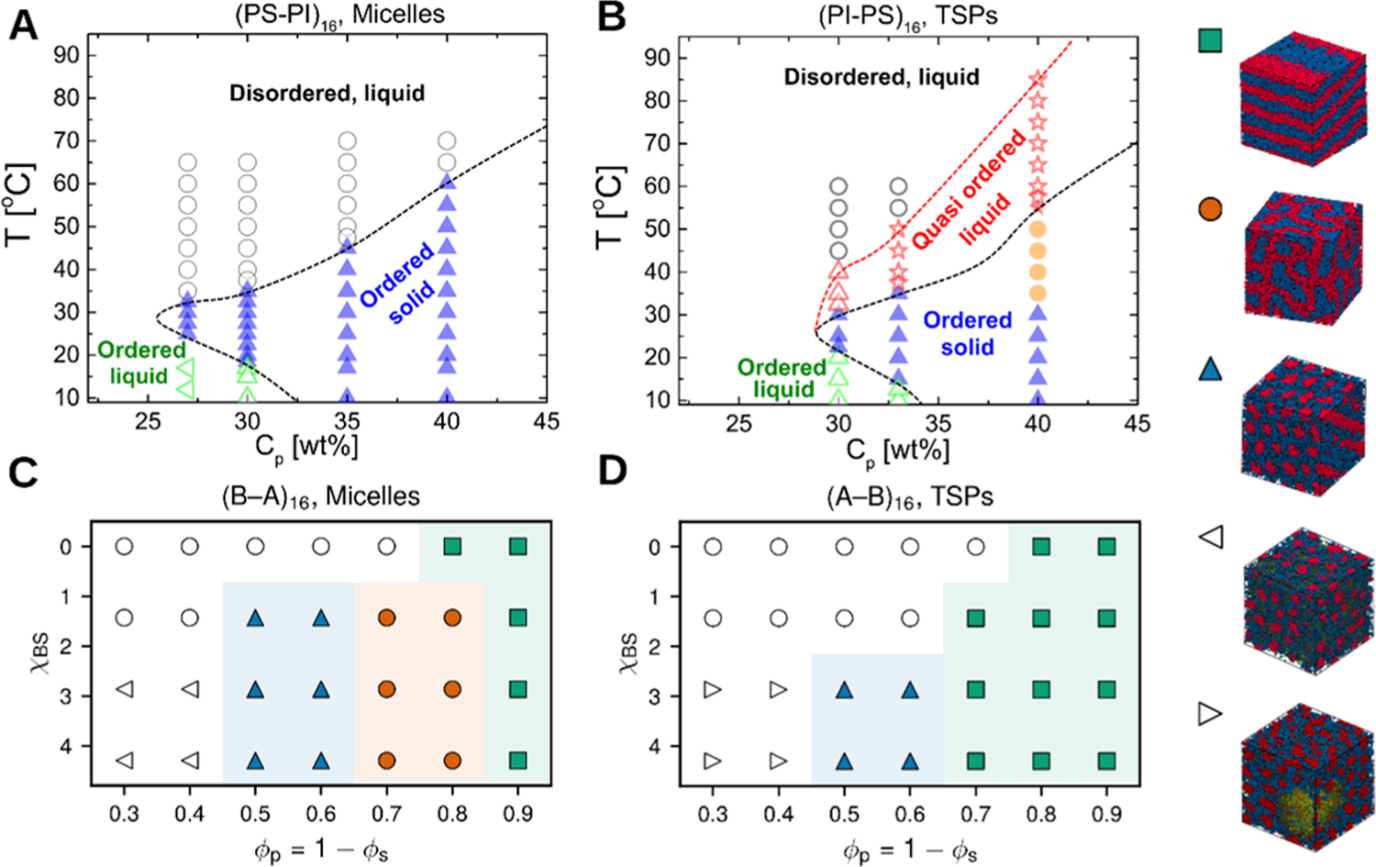
Tentative phase diagrams from experiments and simulations. (A, B): Experimental phase diagrams of the star block copolymer with sticky PS blocks as the inner (A) and outer (B) blocks. The black dashed curve is a guide to the eye, and the region inside corresponds to a solid-like response (closed symbols) characterized by *G*′ > *G*″, whereas the area outside features a liquid-like response (open symbols). The red and black dashed curves in panel (B) define a region of quasi-ordered liquid arrangement at high temperatures. Symbols illustrate the phases obtained from SAXS: disorder (circles), hexagonally packed cylinders (blue and green top-pointing triangles), cubic phase (green left-pointing triangles), textured double diamond (stars), and gyroids (orange circles). (C, D): Phase diagrams in simulations as obtained for model star block copolymers in solution with the inner (C) and outer (D) sticky blocks, as a function of varying incompatibility parameter χ_BS_ and the polymer volume fraction φ_p_. Note that the vertical axes in panels (C, D) are inverted (χ_BS_ values grow from top to bottom) to match the trend with *T* in experiments. The phase diagrams in panels (C, D) were obtained for χ_AS_ = 0, corresponding to experimental phase diagrams (A, B) for *T* > *T*_PI_^Θ^. The snapshots on the right-hand side illustrate the phases obtained in the simulations: lamellae (green squares), gyroids (orange circles), and system-spanning cylinders (blue top-pointing triangles). Partly ordered states with absent long-range order are shown with open triangles: connected bulky cylinders (open left-pointing triangles) and cylinders partly phase-separated from the solvent (open right-pointing triangles; the yellowish spot at the front bottom corner is solvent). Open circles in panels (C, D) highlight disordered system states.

Given the above results and in view of the discussion on the ambiguity associated with the assignment of different ordered phases, we present in Figure 4 a tentative phase diagram deduced from SAXS, rheological, and MDLS experiments for micelles and TSPs, with the aim to compare it against the DPD simulations. In the case of micelles, where the attractive PS block is inside (Figure 4A), at high temperatures, the structure is disordered, and the rheological response is liquid-like. Below a threshold temperature, the inner PS blocks self-assemble into hexagonally packed cylinders. Hexagonal order gives rise to a solid-like rheological response, with transitions in both structure and dynamics taking place at the same temperature. At temperatures below 22 °C, the cloud point of the outer PI block, in the re-entrant melting regime, the ordered liquid structure reflects a mix of cubic and disordered phases. A decrease in star concentration enhances the temperature where re-entrant melting takes place while it reduces the order–disorder transition temperature. In the case of TSPs, where the attractive PS block is outside (Figure 4B), the phase diagram remains qualitatively the same. However, subtle differences exist, reflecting distinct self-assembly of the star block copolymers, and are now discussed. At high temperatures, the structure is disordered, the rheological response is liquid-like, and the microscopic dynamics (relaxation time τ_0_ and exponent *p*) are essentially the same for both systems. The difference between TSPs and micelles appears when they are cooled to temperatures below the disordered state. In TSPs, a quasi-crystalline structure emerges (the region between red and black curves in Figure 4B), which is a textured double diamond structure, a mixture of more cubic phases (for concentrations above 33% w/w, see Figures 2C and S11A), or a weakly ordered hexagonal structure (for concentration below 33% w/w, see Figures S8 and S9). In this regime, contrary to the micellar system, the rheological signal does not turn into a solid-like response, but instead, it remains liquid-like (although, as discussed above, one can still identify distinguishing features compared to the disordered liquid at high temperatures). With a further reduction of temperature, the structure becomes more coherent, and the rheological response turns into a solid-like behavior, similarly to the micellar system but yet with unambiguous differences of microscopic dynamics. Finally, similarly to the micellar system, when the temperature is reduced well below the cloud point of the inner PI block, re-entrant melting takes place. The order–disorder transition takes place at higher temperatures when the attractive PS block is outside. On the other hand, when the PS block is inside, the liquid-to-solid transition occurs at slightly higher temperatures, and the solid-like response extends to lower concentrations.

To support the experimental findings, in Figure 4C,D, we show the observed phases in our simulations of a generic DPD model. To enable simulations of larger systems, we modeled star polymers with shorter arms of length *N* = 10. The χ_AB_-value was adjusted for finite-size effects to match the experimental value (see Section II). In all simulations, we considered *M* = 500 polymer stars at a varying monomer volume fraction φ_p_ = *N*_p_/(*N*_p_ + *N*_s_), where *N*_p_ is the total number of polymer monomers and *N*_s_ is the total number of solvent particles in the system. For a fixed value of φ_p_, we investigated the assembled block copolymer phases as a function of χ_BS_ for χ_AS_ = 0, that is, for good solvent quality for the A blocks and worsening solvent quality for the B blocks. This corresponds to experimental conditions above the Θ-temperature of PI blocks. Finally, note that the goal of these simulations is to explore generic trends in the polymer assembly and its effect on polymer connectivity rather than to systematically evaluate the phase diagrams of copolymers in solution. In any case, the latter is limited by the simplicity of the employed model as well as by the computational cost of DPD simulations with explicit solvent.

While the present simple model of an AB block copolymer reproduces the mean-field phase diagram in the melt (Figure S14 and ref (41)), in a completely neutral solvent (χ_AS_ = χ_BS_ = 0), higher values of the interblock Flory–Huggins incompatibility parameter χ_AB_ are necessary to observe the ordered phase. In a selective solvent, qualitatively increasing the value of χ_BS_ gradually depletes the solvent around the B blocks and causes its partitioning in the A subsystem. This effectively increases the volume fraction of the A blocks and thus leads to distinct block copolymer phases as compared to those obtained for the given block copolymer in the melt.^55^ Based on the above, the purpose of the present simulations is to explore qualitative analogies with the experiments and provide insights for interpreting the findings.

We find a qualitatively similar behavior for the experimental star block copolymers considered in this work. At an intermediate polymer concentration φ_p_ = 0.50–0.60, similarly to experiments, for both micellar and telechelic architectures, we observe the formation of hexagonally ordered cylinders (see Figure 4C,D). In the case of micelles, the cylinders are formed by the star cores stacked on top of each other, whereas in the case of TSPs, they are formed by the associations of the attractive B blocks. In agreement with the experiments, the ordered phase for the micelles onsets at a smaller value of χ_BS_ compared to TSPs (e.g., see the black dashed line in Figure 4A,B). Yet, the observed long-ranged hexagonal order in the simulations is found for a somewhat enhanced polymer volume fraction φ_p_ compared to the experiments, which can be attributed to the simplicity of the simulation model and very short arm lengths of the simulated starts. Other ordered phases emerge upon increasing the polymer volume fraction. While the lamellar phase onsets at φ_p_ = 0.7 for the TSPs (Figure 4D), for the micelles at φ_p_ = 0.70–0.80 and χ_BS_ ≥ 1.43, we find the formation of a gyroid-like phase (see insets in Figure 4), which emerged consistently over multiple independent simulation runs. At a high φ_p_ = 0.9, we find the formation of a lamellar phase for both architectures.

At even lower values of φ_p_ = 0.30–0.40, both micelles and TSPs are partly ordered for χ_BS_ ≥ 2.86, yet the long-range order disappears, and there are some differences between TSPs and micelles. For micelles, we find the formation of cylindrical, nonsystem-spanning bulky aggregates, whereas, for the TSPs, we observe cylindrical aggregates with a fraction of the solvent phase-separated from the polymer component (see snapshots on the right-hand side of Figure 4). Furthermore, we considered the effect of poor solvent quality for both A and B blocks that corresponds to decreasing temperature below *T*_Θ_^PI^ in the experiments at a low polymer concentration (φ_p_ = 0.40). For both architectures at χ_BS_ = 4.23 and χ_AS_ = 2.86, we find macrophase separation between polymeric and solvent components (see Figure S15). We thus attribute the re-entrance regime to the formation of large crystalline aggregates (also probed by SAXS) that are “floating” in the sea of solvent (phase-separated from the solvent, consistent with the re-entrant melting detected in both light scattering and rheological experiments). In summary, while it is not expected that such a simple model system can quantitatively reproduce all details of the experimental phase diagram, the system ordering upon lowering *T*, the assembly of hexagonally packed cylinders at intermediate polymer concentrations, as well as the onset of the cylindrical phase at lower values of χ_BS_ for the micelles, are well captured. It should be noted that the observed phases are not directly related to the patchiness at the single-particle level discussed above.

### III.IV Structure of Hexagonal Phases

**Figure 5:**
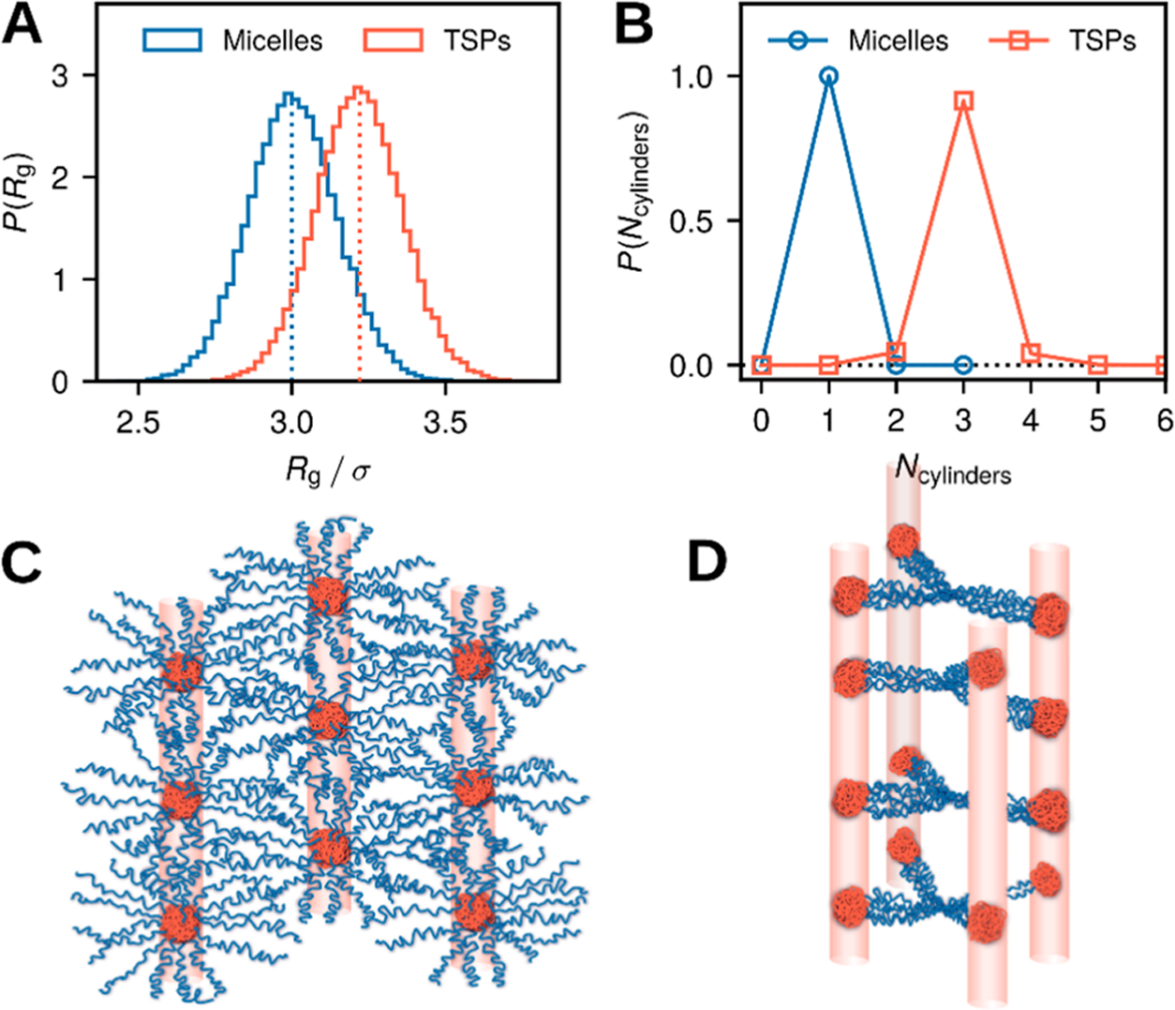
Simulations of the microscopic structure of the star block copolymer network in the phase of hexagonally packed nanocylinders. (A) Distribution of the polymer radius of gyration *R*_g_ for the micellar, (B–A)_16_, and telechelic, (A–B)_16_, star architectures as obtained in simulations. (B) Probability density of finding a star connected to a certain number of cylinders. Schematic illustration of the self-assembled micellar and telechelic star block copolymers, which form interpenetrated (C) and bridged (D) networks, respectively. Typically, at a certain range of concentrations and temperatures, for either situation, these networks are organized in the form of hexagonally ordered nanocylinders (see text). The system configurations used for analysis correspond to φ_s_ = 0.5, χ_AB_ = 9.92, χ_AS_ = 0, and χ_BS_ = 2.86 for both architectures.

Simulations allow us to obtain detailed information about the microstructure of the assembled star polymer network. In Figure 5, we focus on the phase of hexagonally packed nanocylinders that is found in both experiments and simulations at intermediate polymer concentrations (φ_*s*_ ≤ 0.5) for both architectures, TSPs and micelles. Figure 5A shows distributions of the stars’ radii of gyration for TSPs and micelles in the assembled state at φ_*s*_ = 0.5 and χ_BS_ = 2.86. The TSPs feature larger size, with their mean *R*_g_ being about 10% higher than that of the micelles, 3.22(1)σ vs 3.00(1)σ, respectively. This indicates more stretched conformations of TSPs that can potentially distribute their arms into more cylinders. In Figure 5B, we show the probability density of finding a star connected to a number of cylinders in the simulation box (see Section II for details of the analysis algorithm). We find that the micelles exclusively attach to a single cylinder, while the TSPs predominantly connect to three cylinders (a very small fraction of TSPs connected to two or four cylinders is also observed). These observations led us to propose the organization of star polymers schematically illustrated in Figure 5C,D. Although both phases look similar (hexagonally packed cylinders), there are important differences hidden in the microstructure. When the inner block is attractive, the stars’ cores form the cylinder, with the outer blocks forming the shell, akin to grafted colloidal rods. Here, the rods are self-assembled, and their shells are interpenetrated yet disconnected from those of their neighbors (Figure 5C). In stark contrast with the micellar system that forms isolated cylinders, in the system with attractive outer blocks, a single TSP can bridge three cylinders together. Hence, the TSP-ordered nanostructure is a network of interconnected (bridged) cylinders (Figure 5D). Note that this situation of interconnected cylinders is very different from the cylindrical structures formed by ABA linear triblock copolymers. The latter are known to form loops and bridges in a solvent selective for the middle block,^47−49,56^ and there is one intermolecular bridge per molecule. In contrast, our work shows that TSPs form primarily more than one intermolecular bridge per molecule, as the probability of a star belonging to only one cylinder is negligible (Figure 5B). Consequently, in the present case, the network of bridged cylinders is expected to be mechanically stronger. Assuming the absence of pending loops and validity of the affine network model, the data of Figure 2D yield a bridge molar mass of about 25.4 kg mol^–1^ and about 14 bridged arms per star (literally all), a picture consistent with Figure 5B. In addition to the differences in connectivity between the micellar and TSP systems, the cylinder–cylinder distance is slightly smaller for the TSP case, as shown in Figure S16. Note that the intercylinder distance is bridged by one and two stars in the TSP and micellar case, respectively. These microscopic structural differences should have consequences on the rheology of the two phases. In the case of micelles, the relaxation of the stress should proceed hierarchically via PI arm disengagement and then cylinder sliding, and finally, dissociation. On the other hand, for TSPs, there is a strong fixed PI network, such that the system relaxes only after the cylinders dissociate and the arms retract (see also Figure S17 and discussion in the SI). Hence, we expect a different mechanical performance for the two networks. On another note, the formation of three patches for TSPs in concentrated solutions compared to a single patch at the single-molecule level (Figure 1A) indicates the prevalence of intermolecular associations in the former. A final remark concerning the experimental observations and DPD simulations is in order: as indicated in Section II, the star polymers contain very small amounts of impurities (Figure S22). While the big picture indicating tunability of structural and dynamic behavior at different temperatures is not affected, we cannot exclude their influence on the transitions, the unclear order, and the re-entrance upon cooling. On the other hand, impurities at such small quantities should not have a qualitative impact on the results obtained with the simple coarse-grained model utilized here. This aspect deserves a more thorough experimental and simulation investigation in the future.

### III.V Rheological Properties in the Phase of Hexagonally Packed Nanocylinders

**Figure 6:**
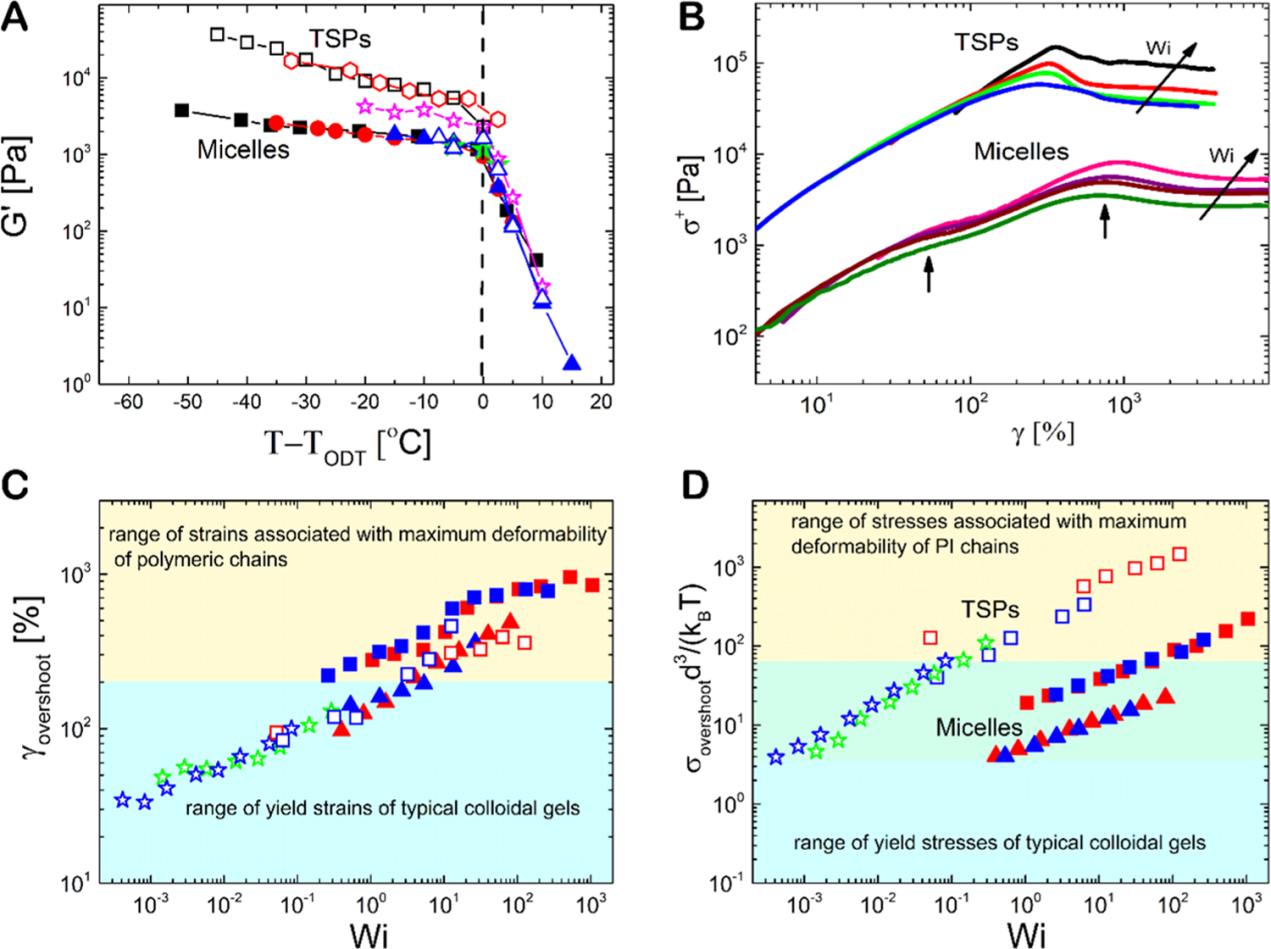
Rheological properties of TSPs and micelles in the concentrated regime. (A) Storage modulus *G*′ measured at a frequency of ω = 1 rad s^–1^ as a function of the distance from the order-to-disorder transition (ODT) for micelles (closed symbols) and TSPs (open symbols) at concentrations of 40%w/w (square symbols), 37% w/w (diamond symbols), 33%w/w (star symbols), 30%w/w (triangle symbols), and 27%w/w (circle symbols). (B) Start-up shear flow experiments performed at different shear rates (various *Wi* numbers), represented as shear stress growth function versus shear strain. The values of *Wi* in TSPs are 6.25 (blue curve), 12.5 (green curve), 31.25 (red curve), and 125 (black curve), and for micelles, they are 52.5 (dark green curve), 105 (brown curve), 210 (purple curve), and 525 (pink curve). In both systems, the star mass concentration is 40% w/w and *T* = 20 °C, well inside the phase of hexagonally packed cylinders. The vertical arrows in panel (B) indicate the positions of the stress shoulder and stress overshoot for the micellar system. (C) Yield strain (taken at the stress peak) and (D) peak of the normalized stress as a function of *Wi* for micelles (closed symbols) and TSPs (open symbols). Symbols in panels (C, D) represent the same concentration as in panel (A). Red, green, and blue symbols denote the data for 20, 25, and 30 °C, respectively. In panel (D), stress has been normalize*d* with *d*^3^/*k*_B_*T*, where *d* is the domain spacing taken from Figure S16. The colored regions in panels (C, D) represent, respectively, the approximate ranges of the strain (at stress overshoot) and the stress overshoot for both linear polymer chains^62^ and colloidal depletion gels.^63^ In panel (D), the ranges of stress overshoot have been calculated for a linear PI solution with the volume fraction of 0.3 using data from the literature.^64^ The light green intermediate zone in panel (D) represents the regime of overlap of stress overshoot for the linear PI chains and colloidal gels.

To test the scenario of Section III.IV, we now examine the rheological properties of TSPs and micelles in the hexagonally packed phases. Figure 6A depicts variations of the storage modulus *G*′ measured at ω = 1 rad s^–1^ as a function of the distance from the order-to-disorder transition (ODT) for both micelles and TSPs at different concentrations (Figure 4). *G*′ decreases upon heating in the ordered state, and when the ODT is crossed, it exhibits a sharp decrease with the data for both systems collapsing in the disordered regime. In contrast, in the ordered regime (*T* < *T*_ODT_), the interconnected nanostructures of TSPs exhibit larger values of *G*′ (up to about 1 order of magnitude) compared to the interpenetrated network in micelles, with steeper temperature dependence. A deeper thermal quench produces a larger difference in *G*′ values between TSPs and micelles.

We also compare the yielding and flow behavior of both networks during start-up in shear. In these experiments, the sample is subjected to an imposed constant shear rate, and the dependence of the shear stress growth function on accumulated deformation is probed. A typical example is shown in Figure 6B at different shear rates for both micelles and TSPs at a concentration of 40% w/w at *T* = 20 °C, well within the region of hexagonally packed cylinders. To compare the two systems, the shear rate γ̇ is normalized with a characteristic relaxation time in the linear regime λ (taken as the inverse frequency at the maximum of *G*″, see the SI for further details), defining a dimensionless Weissenberg number (*Wi* = γ̇λ). For the micellar system, the stress growth function develops a shoulder that is followed by an overshoot and, eventually, a steady state at large accumulated strains. The shoulder appears at a strain value of about 10–15%, similar to typical yield strain values of jammed soft colloidal suspensions when their local caged structure is substantially distorted (here, this corresponds to the distortion of the hexagonal lattice made of packed cylinders). The overshoot occurs at strains of 700–1000%, it increases with *Wi*, and is assigned to the orientation and stretching of PI arms.^57^ For TSPs, there is a single dominant overshoot in the stress growth function, reflecting the breakage of PS patches (cores of nanocylinders), which is associated with stretching of the bridging PI arms and is necessary for yielding and flow.

In general, the bridging of nanocylinders with PS cores in TSPs does not have a significant impact on the yield strain (at overshoot) compared to the interpenetrated structure in the micellar system (Figure 6C). However, it does enhance significantly the yield stress (by more than 1 order of magnitude, Figure 6D), reflecting the stiffening of the network, as evidenced by the increase of the storage modulus. Making soft materials with stronger mechanical coherence without affecting their deformability (being ductile) is a challenge in networks. Usually, there is a trade-off between stiffness and extensibility, i.e., an increase of modulus and stress is linked to lower yield strain and brittleness. The traditional approach of incorporating nanoparticles into polymers (polymer nanocomposites) leads to a trade-off between stiffness and extensibility.^58^ Recent strategies employ complex network architectures such as interpenetrating double and triple networks,^59−61^ where one or more networks play the role of “sacrificial minority network,” whereas the second “majority” network offers large deformability (hence coherence of the overall structure). However, these approaches with complex multicomponent systems have been predominantly used in dry networks. Here, we show that we can achieve the same remarkable rheological properties for solutions and with a much simpler system, a star block copolymer which, depending on solvent–block interactions, may alternate between an interpenetrated (micelles) and an interconnected (TSPs) nanostructure, maintaining the same deformability but increasing mechanical strength.

## IV Concluding Remarks

We have shown that star polymers comprising diblock copolymers, with one of the blocks (either inner or outer) being solvophobic, represent a versatile building block for tunable, soft, patchy colloidal systems. By tuning the strength of attractions (here, solvent quality) through temperature changes, a rich phase behavior emerges, whose most notable effect is the transition from a high-temperature disordered liquid to a low-temperature crystalline structure. The phase diagram exhibits a re-entrant transition where an ordered/solid state can be formed both on heating and cooling, demonstrating the richness of the structural and dynamic behavior of this kind of patchy particles. The bridged nanocylinders in TSPs exhibit coherent organization with enhanced plateau modulus and yield stress compared to interpenetrated micelles or typical colloidal depletion gels, while their yield strain is practically similar. The concept of inverted architecture can be exploited by appropriate tuning of solvent quality, while the number, size, and interactions of blocks control the mechanical response. Therefore, this simple approach offers a promising avenue to produce a new class of responsive materials for diverse applications.

## Supporting Information Available

Dynamic light scattering (DLS) characterization; analysis of SAXS for micelles with 27% w/w; analysis of SAXS for TSPs with 33% w/w at 40 °C; Van Gurp–Palmen representation of rheological data for TSPs with 33% w/w; analysis of SAXS for TSPs with 33% w/w; analysis of SAXS and rheological data for TSPs at 30% w/w; analysis of SAXS and rheological data for TSP with 40% w/w; solvent viscosity at different temperatures; simulations of linear and star block copolymer phases; characteristic size scales from SAXS data for micelles and TSPs; determination of a characteristic time scale of the star block copolymer systems; and details of synthesis and characterization (PDF)
