# Supplementary material for: Interpenetrated and Bridged Nanocylinders from Self-Assembled Star Block Copolymers
Source: Macromolecules. 2024 Jan 30;57(3):926–39. doi: 10.1021/acs.macromol.3c02088 (PMC11190992; doi:10.1021/acs.macromol.3c02088)
Supplement: Supplementary file 1 — ma3c02088_si_001.pdf [file ma3c02088_si_001.pdf]

Supporting Information

Interpenetrated and bridged nanocylinders from self-assembled star block copolymers

Esmaeel Moghimi,[1, 2] Iurii Chubak,[3, 4] Konstantinos Ntetsikas,[5] Georgios Polymeropoulos,[5] Xin Wang,[5] Consiglia Carillo,[1, 2] Antonia Statt,[6] Luca Cipelletti,[7,8] Kell Mortensen,[9] Nikos Hadjichristidis,[5] Athanassios Z. Panagiotopoulos,[10] Christos N. Likos,[3] Dimitris Vlassopoulos*[1, 2]

[1]Institute of Electronic Structure and Laser, FORTH, Heraklion 71110, Crete, Greece

[2]Department of Materials Science and Technology, University of Crete, Heraklion 71003, Crete, Greece

[3]Faculty of Physics, University of Vienna, Boltzmanngasse 5, A-1090 Vienna, Austria

[4]Sorbonne Université CNRS, Physico-Chimie des électrolytes et Nanosystèmes Interfaciaux, F-75005 Paris, France

[5]Polymer Synthesis Laboratory, Chemistry Program, KAUST Catalysis Center, Physical Sciences and Engineering Division, King Abdullah University of Science and Technology (KAUST), Thuwal 23955, Kingdom of Saudi Arabia

[6]Materials Science and Engineering, Grainger College of Engineering, University of Illinois, Urbana-Champaign, Illinois 61801, USA

[7]Laboratoire Charles Coulomb (L2C), University of Montpellier, 34090 Montpellier, France

[8]Institut Universitaire de France, IUF, 75231 Paris Cedex 05, France

[9]Niels Bohr Institute, University of Copenhagen, Universitetsparken 5, 2100 Copenhagen Ø, Denmark

[10]Department of Chemical and Biological Engineering, Princeton University, Princeton, New Jersey 08544, USA

*corresponding author: dvlasso@iesl.forth.gr

Contents

1. Dynamic light scattering (DLS) characterization

2. Analysis of SAXS for micelles with 27% w/w

3. Analysis of SAXS for TSPs with 33% w/w at 40°C

4. Van Gurp – Palmen representation of rheological data for TSPs with 33% w/w

5. Analysis of SAXS for TSPs with 33% w/w

6. Analysis of SAXS and rheological data for TSPs at 30% w/w

7. Analysis of SAXS and rheological data for TSP with 40% w/w

8. Solvent viscosity at different temperatures

9. Simulations of linear and star block copolymer phases

10. Characteristic size scales from SAXS data for micelles and TSPs

11. Determination of a characteristic time scale of the star block copolymer systems

12. Details of synthesis and characterization.

1. Dynamic light scattering (DLS) characterization.

DLS for micelles and TSPs in the dilute regime:

In Fig. S1, we present the intermediate scattering functions (ISFs) at a fixed wavevector value q=24.75μm^{-1} and various temperatures (attraction strengths) in dilute solutions for both micellar (PS is the inner block) and TSPs (PS is the outer block) star block copolymers with the same PS/PI ratio of the arms. When the PS block is the inner block of star, the ISF exhibits two distinct patterns, as seen in Fig. S1A. At high temperature, T = 60°C, the ISF exhibits a nearly single exponential decay, indicating the presence of individual stars freely diffusing in the solution. However, upon cooling the system, the inner PS block becomes attractive and the ISF exhibits a two-step decay. The slow relaxation process becomes more pronounced as T is reduced. It is due to the formation of micellar aggregates of stars through their inner PS blocks. Interestingly, in the case of PS outer block (TSPs), the ISF does not exhibit a slow process in dilute solution (Fig. S1B). This is attributed to the higher probability of intra-star associations for such a high fraction of attractive PS blocks (0.5) which reduces the tendency of TSPs to form inter-star clusters [1–3]. We note that increasing either the TSPs concentration or decreasing the fraction of the outer PS blocks enhances the probability of inter-associations and hence star clustering. For example, in the case of TSPs with PS fraction of 0.5, when the concentration is increased from 1.5% w/w to 2.7% w/w (see Fig. S2A) or when PS fraction is reduced from 0.5 to 0.3 (see Fig. S2B), one can identify in the ISF the emergence of a slow mode indicative of TSPs clustering.

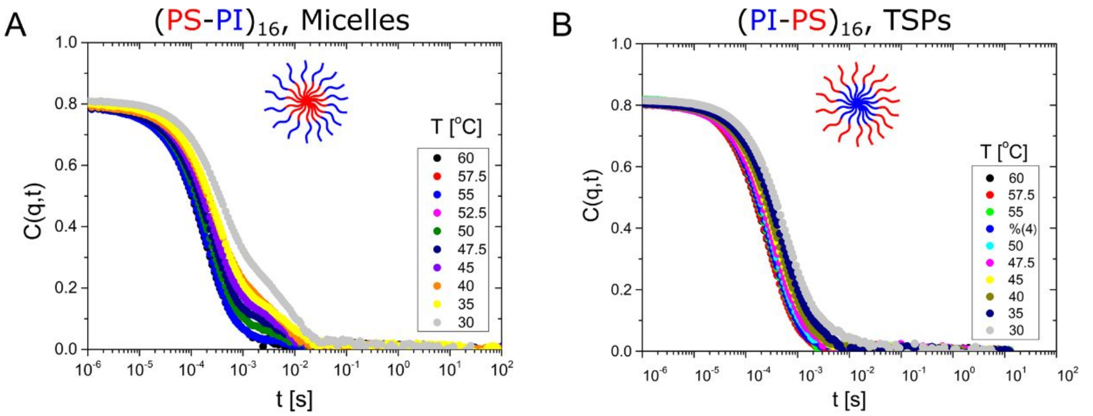

Figure S1. Dynamics of micelles and TSPs in dilute solution: The experimental intermediate scattering functions (ISFs) at different temperatures as shown in the legend, for Micelles (**A**) and TSPs (**B**) with the fraction of the attractive PS block being 0.5. In both (**A**) and (**B**), the star concentration is 1.5% w/w.

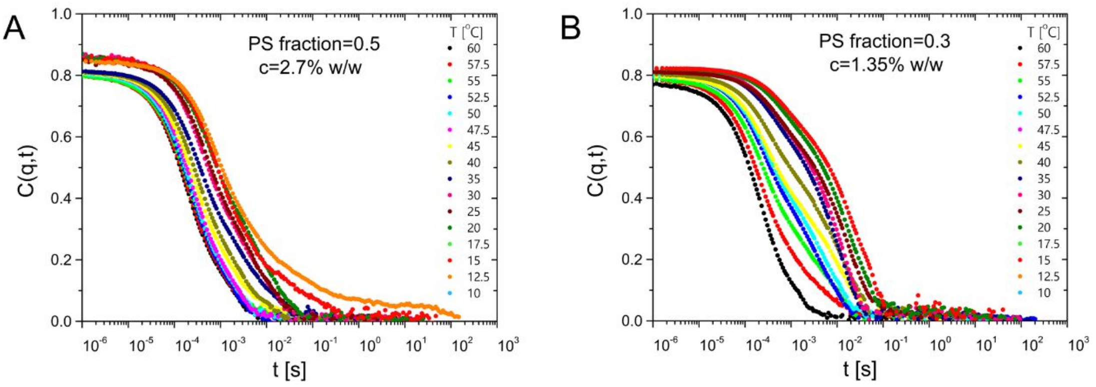

Figure S2. Dynamics of TSPs in dilute solution: The experimental intermediate scattering functions (ISFs) at different temperatures as shown in the legend, for TSPs with the fraction of the attractive PS being 0.5 and concentration 2.7% w/w (**A**) and TSPs with PS fraction of 0.3 and concentration 1.35% w/w (**B**).

Hydrodynamic radius of the individual stars:

To extract hydrodynamic radii associated with the individual stars (fast process in the ISF), the relaxation spectrum is calculated from the inverse Laplace transformation of the ISF using the constraint regularized method discussed in Refs [3,4]. Subsequently, the relaxation time associated with the fast process is used to calculate the diffusion coefficient. The hydrodynamic size of stars is obtained using the Stokes-Einstein-Sutherland relation.

Fig. S3 shows the temperature dependence of normalized R_h for both micelles and TSPs with the same PS/PI ratio of the arms. In both TSPs and micelles, the star size shrinks upon reducing temperature or equivalently worsening the solvent quality. The decay in size initiates at temperatures slightly above the cloud point of the PS blocks (53.5°C), which is expected to be slightly below the PS theta temperature. Hence, the decrease of hydrodynamic size is associated with the collapse of PS blocks. This is consistent with simulation results presented in Fig. 1, as well as findings for the low functionality TSPs, *f*=3 [3,4]. However, in contrast to experiments, simulations (for a single star) show a more collapsed architecture for TSPs compared to micelles. This could originate from a deeper thermal quench in simulations compared to experiments.

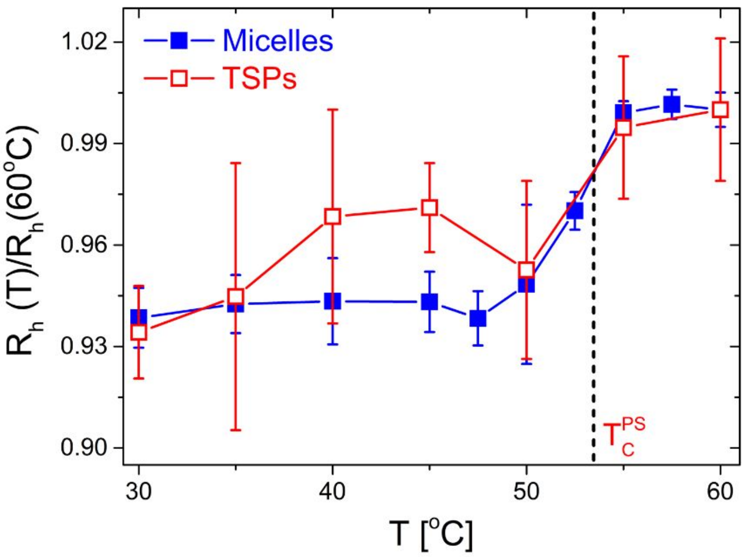

Figure S3. The size reduction of a star block copolymer on worsening solvent quality: Hydrodynamic radius, R_h, of the individual stars, normalized with its value at 60°C in the absence of attractions, as function of temperature. The value of R_h is extracted from the fast process in the experimentally determined ISFs of Fig. S1 for both Micelles (solid blue squares) and TSPs (open red squares). The vertical dashed line indicates the could point of PS block (T_C^{PS}=53.5°C).

2. Analysis of SAXS for micelles with 27% w/w:

In Fig. S4A, we present the change in the peak full width at half maximum of the first-order peak as the function of temperature for the star block with PS as the inner block (micellar case), at 27% w/w concertation. The width of the peak shows a gradual linear decrease on cooling in the disordered regime. However, both the width (Fig. S4A) and the inverse of first-order intensity peak (Fig. S4B) show a drastic decrease when the order to disorder transition (ODT) is reached highlighting that ODT here is a first order transition. In the ordered regime, on the other hand, the width shows a slight decrease on cooling indicating formation of more coherent structures. However, when temperature is further reduced below 20°C (below the cloud point of the outer PI block, 22°C), the width shows a slight but consistent increase which suggests that the structure loses its coherence on cooling below 20°C. However, these slight variations are not unambiguous, given the (weak) uncertainty in the width determination.

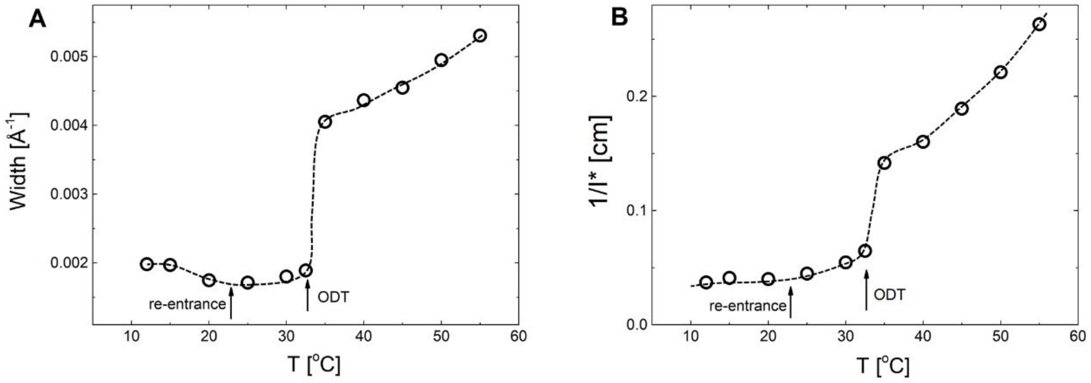

Figure S4. Analysis of SAXS data for micelles with 27% w/w: (**A**) The peak full width at half maximum of the first-order peak and (**B**) the inverse of first-order intensity peak as a function of temperature. The vertical arrows show ODT and re-entrant melting temperatures. The dashed lines in (**A**) and (**B**) are the guide to the eye.

3. Analysis of SAXS for TSPs with 33% w/w at 40°C:

In Fig. S5, we present the SAXS pattern of a star block copolymer with PS as the outer block and 33% w/w concentration at 40°C. The inset shows the original two-dimensional SAXS pattern revealing major texture, likely a result of shear during sample mounting. If we assume cubic symmetry, which is found in numerous polymeric systems, all scattering peaks can be associated to well-defined Bragg peaks with positions relative to the first order peak equal to $\sqrt{2/2}$, $\sqrt{3/2}$, $\sqrt{5/2}$, $\sqrt{6/2}$, $\sqrt{8/2}$, $\sqrt{9/2}$, respectively, corresponding to the cubic reflections [110], [111], [210], [211], [220] and [221]. It is interesting to note that while this sequence of peaks almost ideally resembles the double diamond structure, $Pn\overline{3}m$, with Bragg-peaks [110], [111], [200], [211], [220], and [221], there is a distinct exception: the [200]-reflection in the diamond structure is replaced with a [210]-reflection in the observed spectrum. There is no single cubic structure that fulfills the observed sequence, unless one assumes that some reflections are absent experimentally due to the induced texture. It is interesting to note that the sequence of reflections observed experimentally has previously been observed in a textured sample with cubic structure $Pm\overline{3}m$ based on a NiAl-system [5]. Other possibilities are that the structure is (slightly) non-cubic or a mixture of more (cubic) phases.

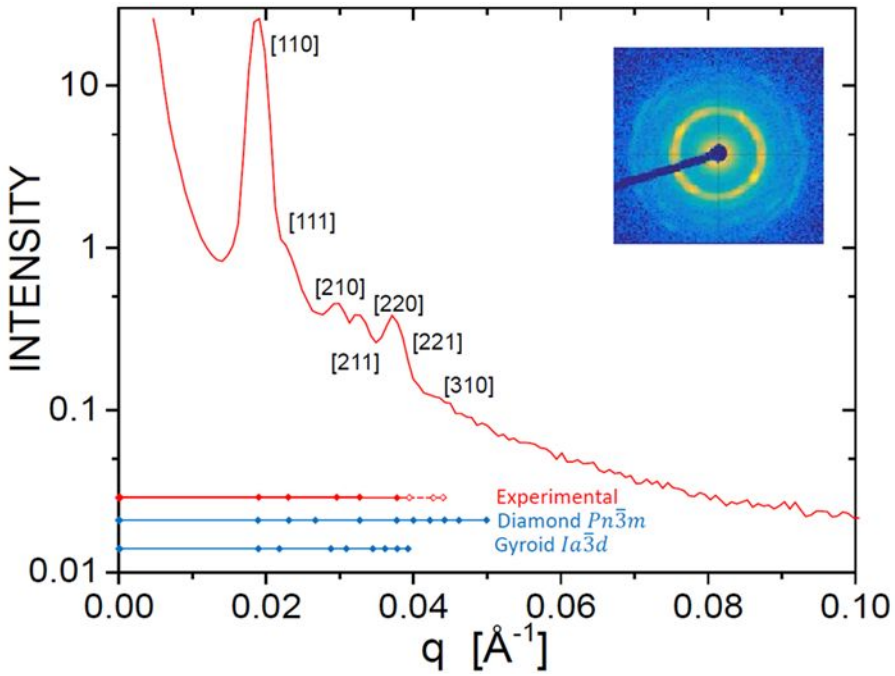

Figure S5. SAXS intensity profile and peaks assignment: Representative SAXS profile at T=40°C for the star block copolymer with PS as the outer block (TSP system) and 33% w/w concentration. The indexes assume cubic symmetry. The horizontal lines with dots refer to experimental peak positions (red line), double diamond and gyroid arrangement (blue lines). The inset shows the two-dimensional SAXS pattern with significant texture.

3. Van Gurp – Palmen representation of rheological data for TSPs with 33% w/w.

In Fig. S6, we present the phase angle $\delta = \text{atan}(G''/G')$ versus the complex modulus G^* (van Gurp-Palmen plot) at various temperatures for the star block with PS as the outer block (TSP system) at a concentration of 33% w/w. At high values of moduli (or equivalently high frequencies), where the local polymeric dynamics is explored, all the data at various temperatures superimpose. The differences are detected at intermediate and lower values of complex moduli. For T>35°C, the phase angle reaches about 90°C at low values of complex moduli which indicates a terminal flow behavior. However, for 37.5°C≤T≤40°C where SAXS shows formation of mixed ordered structures, the phase angle develops a shoulder at intermediate values of complex moduli (3000Pa<G*<10000Pa) which is distinct from the disordered liquid at higher temperatures (for example at T=45°C and 50°C).

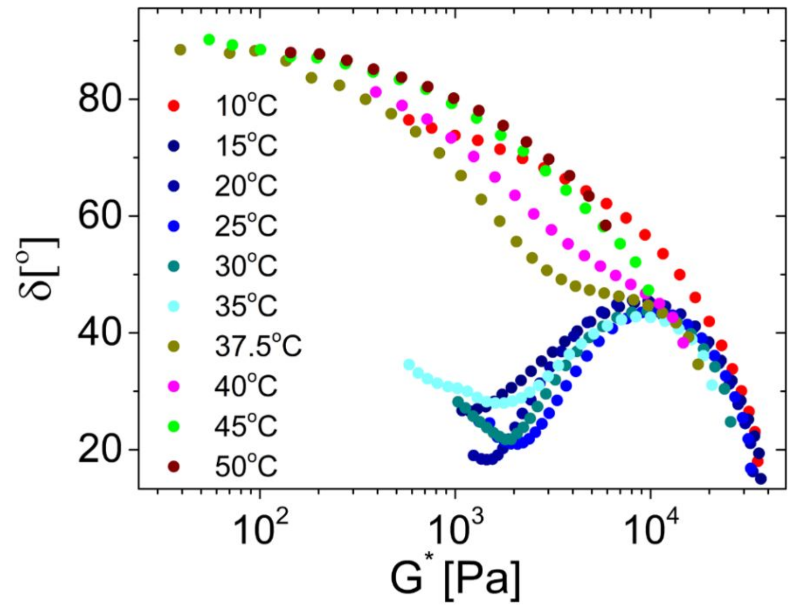

Figure S6. Van Gurp–Palmen representation for TSPs with 33% w/w: The phase angle δ = atan(*G*”/*G*’) versus the complex modulus *G** at different temperatures as marked in the legend.

5. Analysis of SAXS for TSPs with 33% w/w:

In Fig. S7, we show the change in the peak full width at half maximum of the first-order peak as a function of temperature for the star block with PS as the outer block (TSP case) at 33% w/w concertation. In comparison to the micellar system, the width of the peak shows a less significant decrease when ODT is reached. Instead, it exhibits a gradual decrease on cooling. More importantly, when temperature is reduced below 20°C (below the cloud point of inner PI block, 22°C), the width shows a consistent and unambiguous increase on cooling, which suggests that structure loses its coherence on cooling below 20°C. This clearly differs from the respective data at 30%w/w presented in the next section.

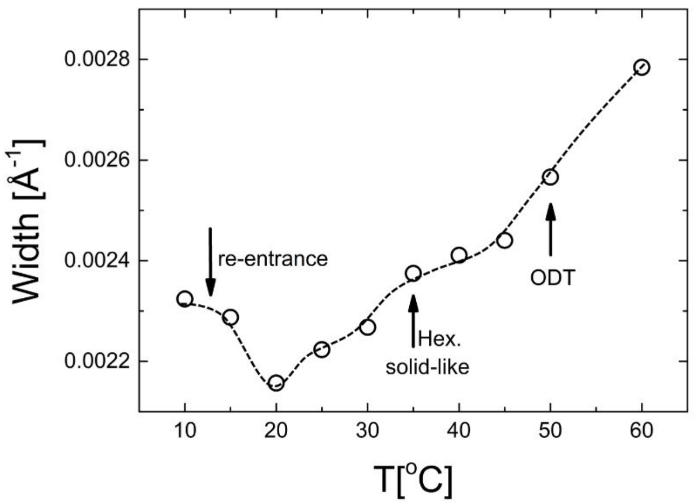

Figure S7. Analysis of SAXS data for TSPs with 33% w/w: The peak full width at half maximum of the first-order peak as a function of temperature. The vertical arrows show the ODT (formation of texture with double diamond structure), formation of hexagonal structure with solid-like rheological response and re-entrant melting temperatures. The dotted line is a guide to the eye.

6. Analysis of SAXS and rheological data for TSPs at 30% w/w:

In this section, we present changes in the structural and rheological properties on varying the temperature for the system in which the attractive PS block is outside (TSP system). The results are presented in Fig. S8 for the star block with a concentration of 30% w/w (see Fig. 2, C and D of the main manuscript for data at a concentration of 33% w/w). At high temperatures of 50°C-60°C, the structure is disordered although some weak short-ranged correlations are detected. In this regime, rheology detects a well-defined liquid-like response (Fig. S8B), a result further supported by simulations (see text and discussion and Fig.S15 below). On cooling to 40°C, SAXS shows a transition to a hexagonal structure, but still with a relative broad first order Bragg-peak and with only few higher order Bragg-peaks ($\sqrt{3}$-peak), indicating limited correlated structure. This is better seen by looking into the peak full width at half maximum of the first-order and the inverse of first-order intensity peak as a function of the temperature. In the case of micellar system (PS-inner block), both the peak broadness and the inverse of first-order peak shows a sharp decrease at ODT (see Fig. S4). In contrast, for the TSP, this drop is rather gradual highlighting the fact that at the ODT the structure is not yet fully ordered (Fig. S9). In this case, rheology still shows liquid-like response (see Fig. S8B). However, for 32.5°C≤T≤40°C, rheology exhibits a distinct dynamics at higher frequencies (see Fig. S8B) which could be due to a pre-transition to a different state. This is better seen in van Gurp-Palmen representation (Fig. S10) where for 32.5°C≤*T*≤ 40°C, one can identify a shoulder at intermediate values of the complex modulus, 400Pa<G*<2000Pa, in agreement with the presence of degree of order in the structure.

A further cooling to 30°C leads to a clear solid-like response (Fig. S8B). Moreover, the values of the moduli at low frequencies fairly follow a power law with exponent 1/3, which is the rheological signature of hexagonal order [6,7]. Below T=30°C, the scattering shows narrow Bragg-peaks, and additional higher order peaks ($\sqrt{4}$- and $\sqrt{7}$-peaks), indicating more correlated structure, in agreement with the stronger solid-like response at these temperatures.

The sample re-melts when it is cooled to 20°C, which is below the cloud point of the inner PI block, 22°C (Fig. S8B). However the data indicate a broad relaxation spectrum and the terminal response is barely reached at the lowest frequencies; this suggests the presence of internal structural relaxation (also evidenced in the van Gurp-Palmen representation of Fig.S10) and is consistent with the MDLS data. In this case, the structure remains hexagonal. The additional $\sqrt{9}$-peak may indicate a form factor that reaches out to larger q-values indicating smaller scattering objects (smaller cores). This is in agreement with the fact that the solvent is excluded from the inner PI-blocks below 22°C. On the other hand, a slight growth of the peak broadness is seen for T<15°C (Fig. S9A), again not fully unambiguous. This trend is consistent with the structure becoming less coherent on cooling below 15°C, likely due to smaller coherent domains as indicated in the simulations. This leads to the liquid-like character.

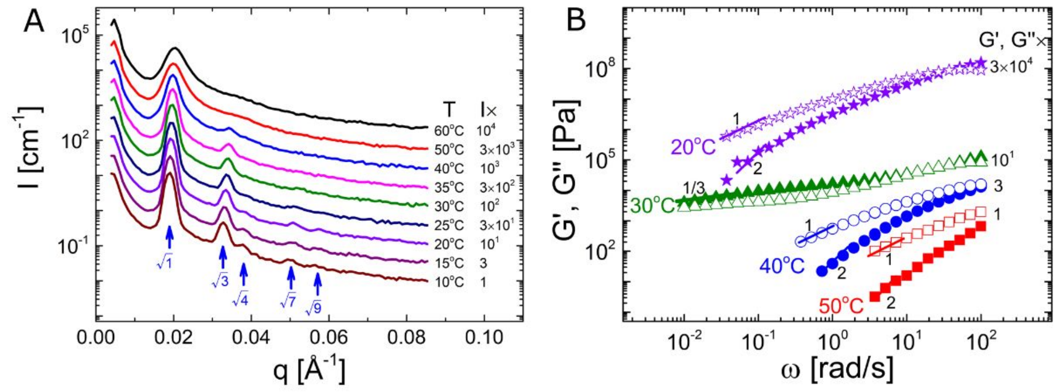

Figure S8. Structural and rheological properties of TSPs with 30% w/w: (**A**) Representative SAXS profiles and (**B**) Dynamic frequency spectra depicting the storage G' (closed symbols) and loss G'' (open symbols) moduli as a function of angular frequency at different temperatures (shown in the legend). The arrows with numbers in (**A**) refer to the relative positions for the first few allowed reflections for the hexagonal order. The solid lines in (**B**) show the low frequency power law slope of moduli. For clarity, values of intensity and viscoelastic moduli are shifted vertically as marked in the legend.

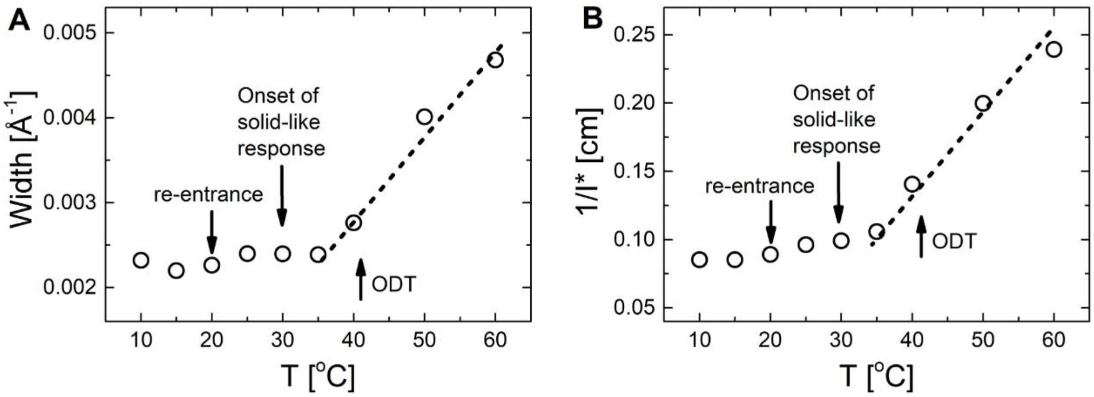

Figure S9. Analysis of SAXS data for TSPs with 30% w/w: (**A**) The peak full width at half maximum of the first-order peak and (**B**) the inverse of first-order peak as a function of temperature. The vertical arrows show ODT, the onset of solid-like response and re-entrant melting temperatures. The dashed lines in (**A**) and (**B**) are the guide to eyes and highlight the fact that upon cooling, the system keeps the same trend (width and intensity of the first order-peak) as in the disordered phase even down to a few degrees below the ODT, well in the ordered phase (see the text for detailed discussion).

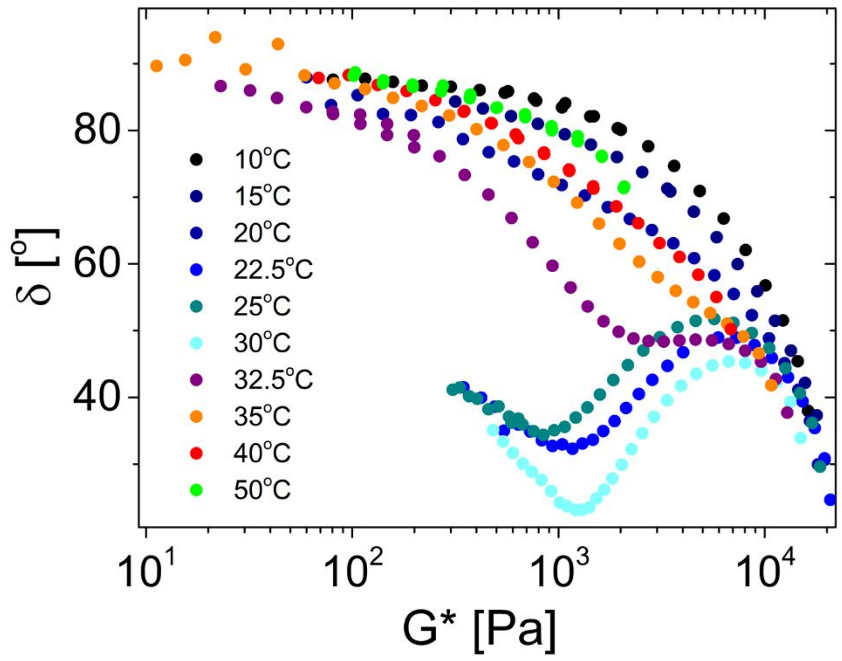

Figure S10. Van Gurp–Palmen representation for TSPs with 30% w/w: The phase angle δ versus the complex modulus G* at different temperatures as shown in the legend. For 32.5°C≤T≤ 40°C, a shoulder is detected at intermediate values of complex modulus (400Pa<G*<2000Pa).

7. Analysis of SAXS and rheological data for TSP with 40% w/w:

In this section, we present changes in the structural and rheological properties on varying the temperature for the system in which the attractive PS block is outside (TSP), at a higher concentration of 40%. For T >85°C, the structure seems to be disordered (Fig. S11A), although similar to the case of the system at a concentration of 33% one can still identify weak intensity peaks indicating a transition regime from truly disordered to ordered structure. For 50°C≤T≤85°C, SAXS shows a transition to a structure with basically the same characteristics Bragg-peaks observed in the 33%-sample, where we speculated that these features may correspond to a texture with double diamond structure or a (slightly) non-cubic structure or a mixture of more (cubic) phases. However, the rheological response for this range of temperature is rather rich and complex: In this regime, rheology identifies liquid-like response although a terminal flow has not reached (see for example DFS at T=75°C). Moreover, for 50°C≤T≤ 65°C, the van Gurp-Palmen plot shows the presence of a shoulder at intermediate values of complex modulus or equivalently intermediate frequencies (Fig. S12). An interesting critical gel-like response emerges at T=55°C where at low frequencies both elastic and viscous moduli are nearly equal and follow a slope of 0.5 (Fig. S11B). However, a carful inspection of SAXS spectra reveals an interesting change on cooling. There is a shoulder/peak at $q/_{q^*} = \sqrt{3/2}$ associated to a double diamond structure. The intensity of this peak exhibits a weak but consistent decrease on cooling. This may suggest that the double diamond structure is disappearing on cooling. For T<50°C, the shoulder at $q/_{q^*} = \sqrt{3/2}$ completely disappears. Moreover, the weak peak at $q/_{q^*} = \sqrt{10/2}$ which also represents the double diamond structure

disappears. On the other hand, rheology shows the development of a well-defined plateau elastic modulus at low frequencies (see for example DFS at T=40°C in Fig. S11B) which is a rheological signature of gyroid or other cubic structures [8–16].

The structure turns into a hexagonal one when temperature is reduced below 30°C. However, within the range of probed frequencies, the low frequencies values of viscoelastic moduli do not follow the power law slope of 1/3 which is the rheological signature of hexagonal order [6,7]. Such scaling of moduli may take place at much lower frequencies, outside the experimental time window

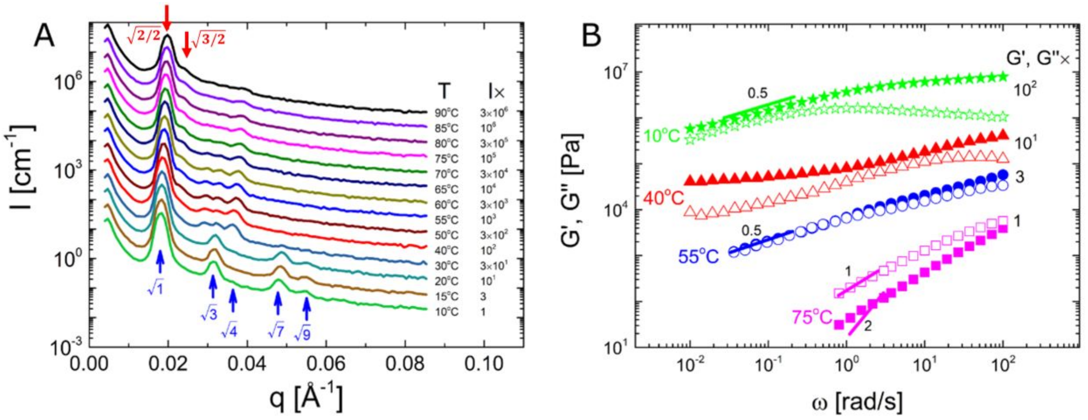

Figure S11. Structural and rheological properties of TSPs with 40% w/w: (**A**) Representative SAXS profiles and (**B**) Dynamic frequency spectra depicting the storage G' (closed symbols) and loss G'' (open symbols) moduli as a function of angular frequency at different temperatures (shown in the legend). The blue arrows with numbers in (**A**) refer to the relative positions for the first few allowed reflections for the hexagonal order and the red arrows show the position of first peak and the $q/_{q^*} = \sqrt{3/2}$ associated to double diamond structure. The solid lines in (**B**) show the low frequency power law slope of moduli. For clarity, values of intensity and viscoelastic moduli are shifted vertically as marked in the legend.

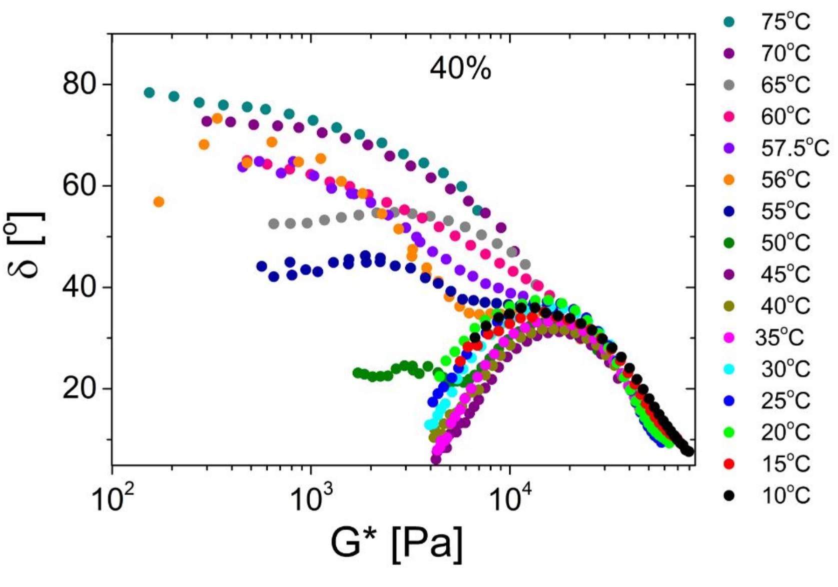

Figure S12. Van Gurp–Palmen representation for TSPs with 40% w/w: Phase angle δ versus the complex modulus $G*$ at different temperatures as shown in the legend.

8. Solvent viscosity at different temperatures:

Fig. S13 shows the change of solvent viscosity with temperature.

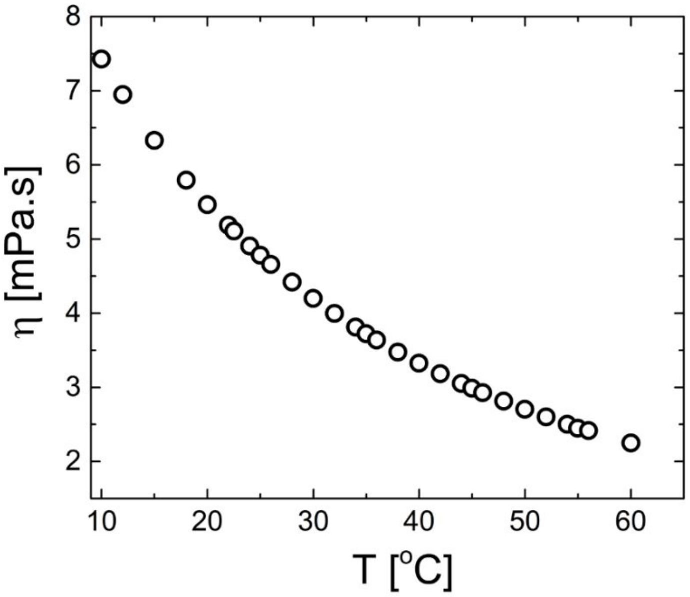

Figure S13. Solvent viscosity: The viscosity of 1-phenyldodecane (solvent) as a function of temperature.

9. Simulations of linear and star block copolymer phases:

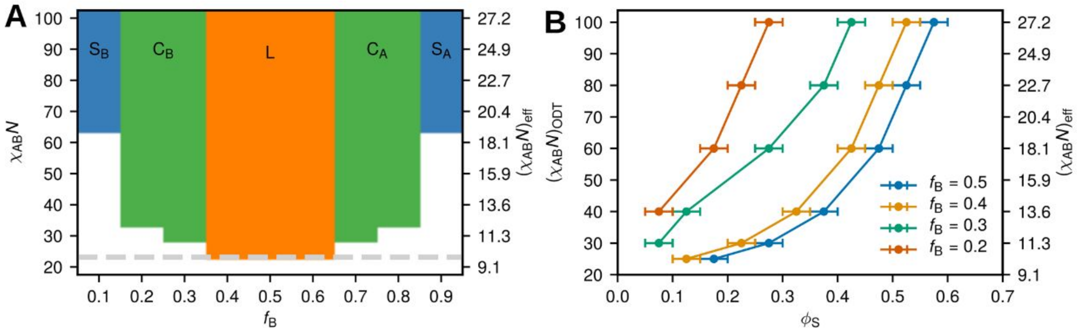

Figure S14. Phase diagrams from simulations: (A) The phase diagram of linear AB diblock copolymers of length $N = 10$ in the melt. $f_B = N_B/N$ indicates the relative length of the B block. The observes phases include spheres (S), cylinders (C), and lamellae (L). The gray dashed line is the mean-field value of $\chi_{AB}N$ at the order-disorder transition for $f_B = 0.5$. (**B**) Value of $(\chi_{AB}N)_{ODT}$ for diblock AB copolymer dissolved in a neutral solvent ($\chi_{AS} = 0$, $\chi_{BS} = 0$) at the solvent volume fraction ϕ_S for different f_B.

In Fig. S15, we show the self-assembly of stars at the polymer concentration of $\phi_{\mathrm{p}} = 0.40$ and poor solvent quality for both A and B blocks with $\chi_{\mathrm{BS}} = 4.23$ and $\chi_{\mathrm{AS}} = 2.86$. This resembles a decreasing temperature below T_{Θ}^{PI} in the experiments. Such conditions lead to the phase separation between the polymer and solvent components. Since the solvent is very poor with respect to two blocks, it is almost completely expelled from the polymer and hence the rich polymeric phase shows a lamellar structure similar to that found in the melt or highly concentrated solution of stars (see Fig. 4). For a better solvent for A blocks, other phases can be present.

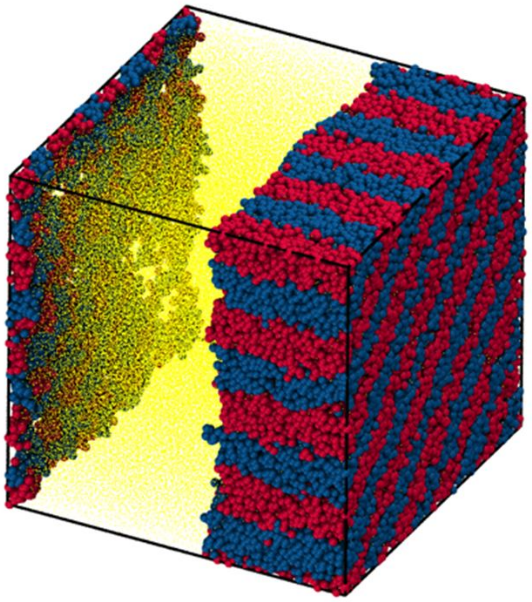

Figure S15. Both blocks subject to bad solvency conditions: Phase separation between the polymer and solvent components for the TSP system with $\chi_{\mathrm{AS}} = 2.86$, $\chi_{\mathrm{BS}} = 4.23$, and $\chi_{\mathrm{AB}} = 9.92$ at the polymer concentration $\phi_{\mathrm{P}} = 0.4$. A blocks are blue, B blocks are red and solvent particles are yellow. The phase separation was observed consistently over three simulation runs. Qualitatively similar behavior was found for the micellar architecture at the same system parameters.

10. Characteristic size scales from SAXS data for micelles and TSPs:

In Fig. 16, we show the variations of the domain spacing with temperature for both micellar and TSP systems at different concentrations. In both systems, a decrease of temperature increases the domain spacing. An increase in stars concentration reduces the domain spacing. Finally, at the same concentration and temperature, the domain spacing in micelles is larger compared to TSPs.

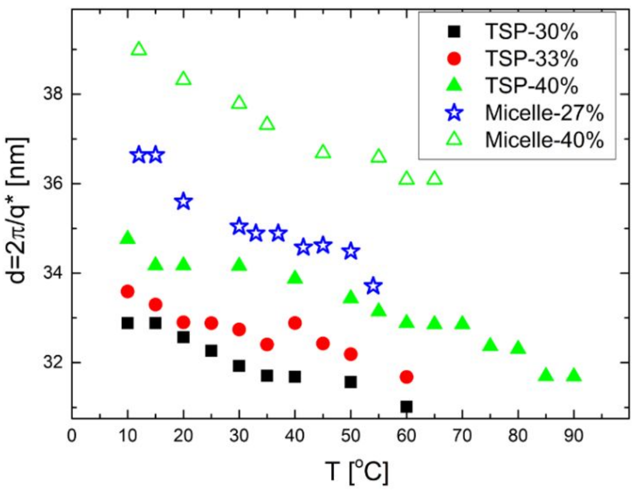

Figure S16. Characteristic size scales from SAXS: The lattice spacing as a function of temperature at 11 in the legend. Closed symbols are TSPs (PS outside) and open symbols are micelles (PS inside).

11. Determination of a characteristic time scale of the star block copolymer systems:

Here, we present arguments to support the choice of the inverse of frequency at the maximum of G" as a characteristic time scale of the experimental systems. In this context, we first show in Fig. S17 indicative linear viscoelastic master curves (moduli G' and G'' plotted against reduced angular frequency $a_T\omega$) at a concentration of 40% w/w and various temperatures below the order-to-disorder transition, for both the micellar and TSP systems. Note that the viscoelastic moduli at various temperatures were shifted only along the frequency axis to obtain the master curves.

The G' values for micelles exhibit a plateau at high frequencies which is associated to the weakly entangled interpenetrated of PI chains, which decreases logarithmically as the (shifted) frequency decreases. This broad range of weak relaxation is typical of grafted arms conventional star polymers [17] and likely further promoted here by the spatial fluctuations of the ordered nanocylinders. On the other hand, values of viscoelastic moduli at lower frequencies (below 0.1 rad/s) follow a stronger, power-law decrease with exponent of about 1/3, conforming to the sliding of hexagonally packed nanocylinders (with PS cores) [6,7]. The (inaccessible) terminal regime should be related to the melting of the nanocylinders and the center of mass of the individual stars. In the case of TSPs, the viscoelastic spectra is very different, with G' exhibiting two pseudo-plateau regions: the high-frequency apparent plateau which is attributed to the network of bridged PI chains (with larger modulus compared to physical entanglements) reinforced by the presence of PS domains as described in Ref. [18]. On the other hand, the low-frequency plateau is associated to microphase-separated nanocylinders which eventually should slide at lower frequencies outside the experimentally accessible window. The transition between the two pseudo-plateau regions, over a small frequency range, conforms to a Rouse-like power-law decay (slope of 1/2) and should reflect the local fluctuations of bridged PI chains due to local motion of the PS patches which

remain in phase separated but can slightly move since PS is plasticized at the examined conditions. It is important to note that even in the disordered state, TSPs exhibit weak short-ranged structural correlations (see Fig. S11A) which indicates the presence of the inter-associations between the outer PS blocks. In contrast, in micelles the outer PI blocks are interpenetrated. Hence, the larger values of the high frequency plateau in TSPs compared to micelles could originate from the bridged nanostructure [7].

Based on the picture provided above, it is possible to divide the viscoelastic spectra of both TSPs and micelles into two parts, separated by a critical frequency ω_c, corresponding to a (broad) peak in G''. For $\omega > \omega_c$, chain dynamics associated with network formation dictate the linear rheological response, whereas for $\omega < \omega_c$, the collective (microphase separated PS domains) dynamics dominate. Hence, the inverse of ω_c can be used as the characteristic time scale of the system which represents the transition time from polymeric network to collective dynamics of the nanocylinders.

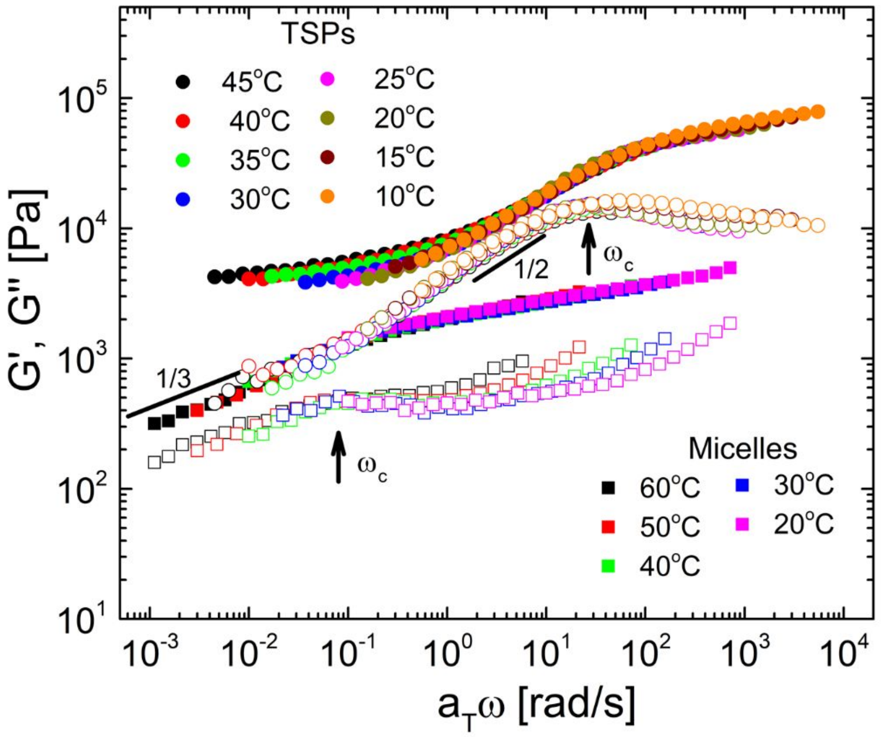

Figure S17. Linear rheology in the phase of hexagonally packed cylinders: Master curves of elastic G' (closed symbols) and loss G'' (open symbols) moduli as a function of the shifted angular frequency. Circle symbols represent TSPs (PS outer block) whereas square symbols show the micellar system (PS inner block). In both systems the star concentration is 40% w/w and the reference temperature is $T_{ref} =$ 40°C ($a_T(T_{ref} = 1)$).

12. Details of synthesis and characterization.

Materials:

Vinylmagnesium bromide solution (1.0 M in THF, Aldrich), tetravinylsilane (97%, Aldrich), dichloromethylsilane (> 97%, Aldrich), and platinum(0)-1,3-divinyl-1,1,3,3-tetramethyldisiloxane complex solution (in xylene, Pt ~2 %, Aldrich) were used as received. Tetrahydrofuran was distilled over Na, then degassed three times and stored in the glovebox for use. Styrene (Sigma-Aldrich, 99%) was purified *via* successive distillations over CaH_2 (Sigma-Aldrich, 95%) and dibutyl-magnesium (1 M in heptane, Sigma-Aldrich) and stored at -20°C in pre-calibrated ampoules. Isoprene (Sigma-Aldrich, 99%) was purified via successive distillations over CaH_2 and *n*-BuLi, at -10°C, and stored at -20°C in pre-calibrated ampules. Benzene (Sigma-Aldrich, 99.8%) and methanol (terminating agent, Sigma-Aldrich, 99%) were purified according to the standards required for anionic polymerization, using well-established high-vacuum procedures.[19] *sec*-Butyllithium (*s*-BuLi, 1.4 M in cyclohexane, Sigma-Aldrich) was diluted with purified benzene. The polymerization and linking reactions were performed under high vacuum conditions in sealed custom-made glass reactors equipped with break-seals and constrictions.

Instrumentation for synthesized material characterization

Size exclusion chromatography (SEC) measurements were carried out at 35°C through a Viscotek GPCmax VE-2001 with THF as the eluent at a flow rate of 1.0 mL/min, equipped with an isocratic pump, Styragel HR2 and HR4 columns in series (300 mm × 8 mm) and a differential refractive index detector (DRI). The system was calibrated with polystyrene (PS) standards (M_p: 370 to 4,220,000 g/mol). Triple-detection measurements were carried in the same instrument, which was also equipped with a two-angle light scattering detector (15° and 90°, λ = 658 nm) and a viscometer. These measurements were used to calculate the weight-average molecular weight (M_w) and polydispersity index (Đ) of the linear and final star block copolymers. Proton nuclear magnetic resonance spectroscopy (^{1}H-NMR) spectra were recorded at a Bruker AVANCE III spectrometer operating at 500 MHz. Chloroform-*d* ($CDCl_3$) was the solvent at room temperature.

Synthesis of the coupling agent (2G-Cl)

A linking agent with 16 chlorosilane bonds was synthesized using tetravinylsilane as the initial core molecule, methyldichlorosilane as the propagating unit, and vinylmagnesium bromide for the transformation of each silicon chloride to vinyl group [20]. In general, two reactions were involved in the synthesis of each generation: hydrosilylation of vinylsilane with dichloromethylsilane and nucleophilic replacement of silicon chloride by vinylmagnesium bromide. A general synthetic scheme is presented in Fig. S18.

Synthesis of 1G

2.7 g (20 mmol) tetravinylsilane, 10.1 g (88 mmol) dichloromethylsilane, 4 drops of platinum(0)-1,3-divinyl-1,1,3,3-tetramethyldisiloxane complex solution and 40 mL

anhydrous THF was added into a 250 mL flask equipped with a condenser. Then, the reaction was carried out in a 50°C oil bath. The oil bath was removed (sometimes a water-ice bath was needed) as soon as the reaction solution started to reflux (exothermic reaction). The temperature was controlled to let the exothermic reaction go smoothly. After the exothermic period, the reaction was kept under 50°C for 4 h. Then, the reaction flask was cooled to room temperature; the reaction flask was then connected to a vacuum system to remove the excess of dichloromethylsilane and THF. After that, the flask was refilled with Argon, and 40 mL of fresh anhydrous THF was transferred to the flask. The prepared solution was added dropwise into a vinylmagnesium bromide solution (192 mL, 192 mmol, 1M in THF). The mixture was stirred at room temperature overnight. Afterward, 200 mL of hexane was added, and the mixture was washed with 200 mL of water three times and with saturated NaCl solution twice. The organic solution was dried over anhydrous $MgSO_4$. The crude product was obtained after the removal of the solvent. The product was further purified by a silica gel column chromatography eluted with a solution of ethyl acetate/hexane = 1/50 to give a pure oily product (1G, Fig. S18A).

Synthesis of 2G-Cl

2.6 g of the synthesized 1G (5 mmol), 5.1 g (44 mmol) of dichloromethylsilane, 4 drops of platinum(0)-1,3-divinyl-1,1,3,3-tetramethyldisiloxane complex solution, and 40 mL anhydrous THF was added into a 250 mL flask equipped with a condenser. Then, the reaction was carried out in a 50°C oil bath. The oil bath was removed as soon as the reaction solution started to reflux (exothermic reaction). The temperature was controlled to let the exothermic reaction go smoothly. After the exothermic period, the reaction was kept under 50°C for 4 h. Then, the reaction flask was cooled to room temperature; the reaction flask was connected to a vacuum system to remove the excess dichloromethylsilane and all THF. The obtained 2G-Cl was stored in the glovebox for further use.

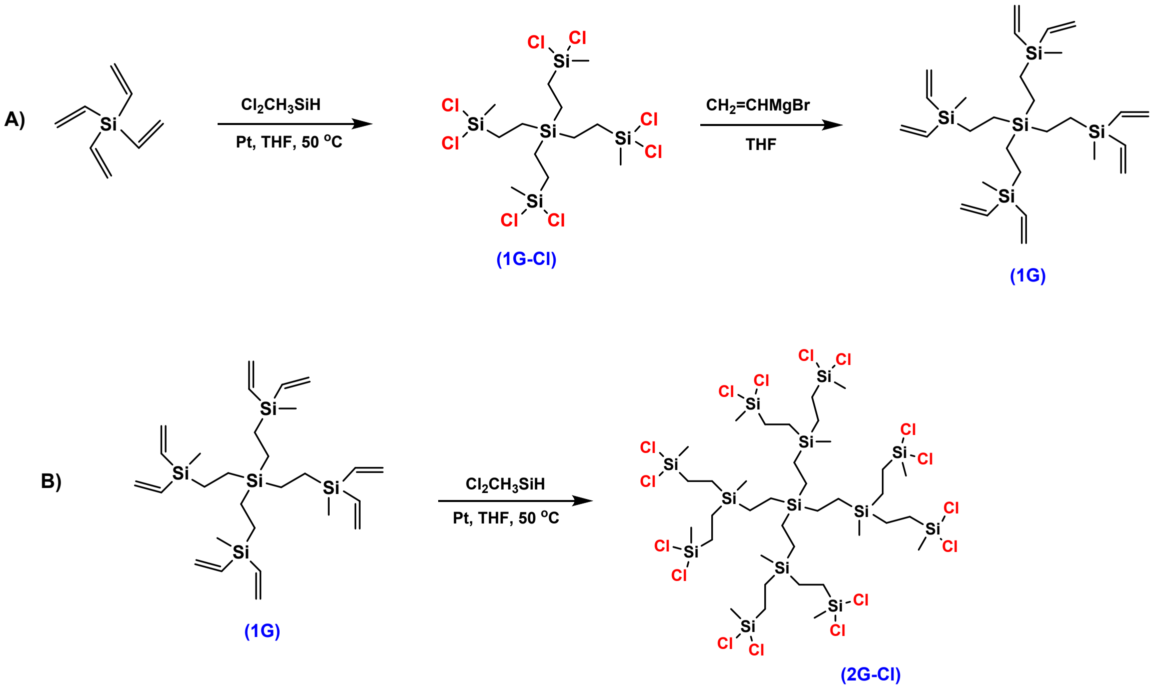

Figure S18. General procedure for the synthesis of the linking agent with 16 chlorosilane bonds (2G-Cl).

Synthesis of 16-arm star block copolymers [(PS-*b*-PI)$_{16}$ and (PI-*b*-PS)$_{16}$]

A typical procedure for the synthesis of the (PS-*b*-PI)$_{16}$ star block copolymer is as follows. Styrene (2.36 g) was polymerized at room temperature, using *sec*-butyllithium (0.094 mmol) as the initiator and benzene as the solvent. The mixture was left to react for 18 hours and then an aliquot was taken by heat-sealing the corresponding constriction tube for molecular characterization (SEC). Afterward, the appropriate amount of isoprene (2.36 g) was added to the reaction mixture, and the polymerization was left until completion. After 24 hours an aliquot was taken containing ~ 0.5 g of the linear block copolymer for molecular characterization. The remaining "living" polymer solution was reacted with the linking agent (2G-Cl, 0.004 mmol) to form the 16-arm star diblock copolymer. An excess of ~ 30% of the "living" linear diblock copolymer was used to drive the reaction to completion. The linking reaction was monitored by SEC and lasted for 3-4 weeks. After the completion of the reaction, the excess of the living chains was terminated by adding degassed methanol and the solution precipitated in a large amount of methanol. The 16-arm star diblock copolymer (PS-*b*-PI)$_{16}$ was purified from the unreacted linear chains by repeated solvent/non-solvent (toluene/methanol) fractionations. The synthetic strategy is presented in Fig. S19A. A similar procedure was followed in the case of (PI-*b*-PS)$_{16}$ star block copolymer, where isoprene was polymerized first followed by the addition of a small quantity of THF and subsequent addition of styrene (Fig. S19B). The molecular characteristics of the linear diblock copolymers and the final 16-arm star block copolymers are presented in Table S1. All intermediates and final products were analyzed by SEC and ^{1}H-NMR spectroscopy (Figs. S20-S23).

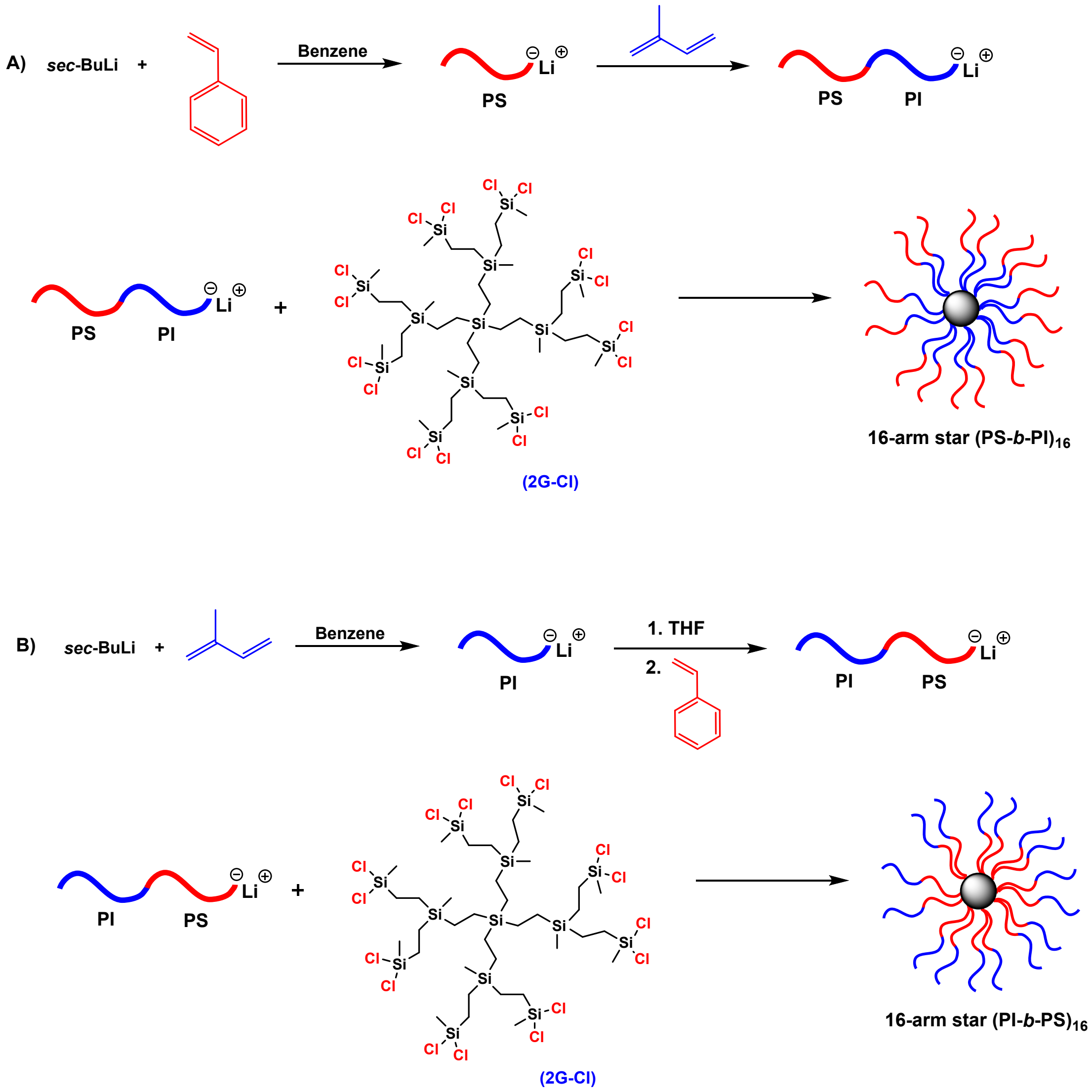

Figure S19. General reaction schemes for the synthesis of 16-arm star block copolymers: (**A**) 16-arm (PS-*b*-PI)$_{16}$ and (**B**) (PI-*b*-PS)$_{16}$.

Molecular characterization of the linear diblock copolymers and the corresponding 16-arm star block copolymers

Table S1. Molecular characteristics of linear PS-*b*-PI, PI-*b*-PS, and the corresponding final 16-arm star diblock copolymers [(PS-*b*-PI)$_{16}$ and (PI-*b*-PS)$_{16}$].

Sample	$\bar{M}_n^{PS}$ [a] (g/mol)	$\bar{M}_n^{PI}$ [b] (g/mol)	$(\bar{M}_w^{star})_{LS\text{-}SEC}$ (g/mol)	$(\bar{M}_w^{linear})_{LS\text{-}SEC}$ (g/mol)	$Đ^{SEC}$ (star)	$f_{(PS)}$[b]	$f_{(PI)}$[b]
**(PS-*b*-PI)$_{16}$**	26,000	27,000	835,200	55,300	1.11	0.49	0.51
**(PI-*b*-PS)$_{16}$**	25,000	26,000	-	-	1.09	0.49	0.51

[a]The M_n was calculated by SEC in THF at 35°C calibrated with PS standards, [b]M_n and mass fractions were calculated via ^{1}H-NMR spectroscopy in $CDCl_3$ at 25 °C.

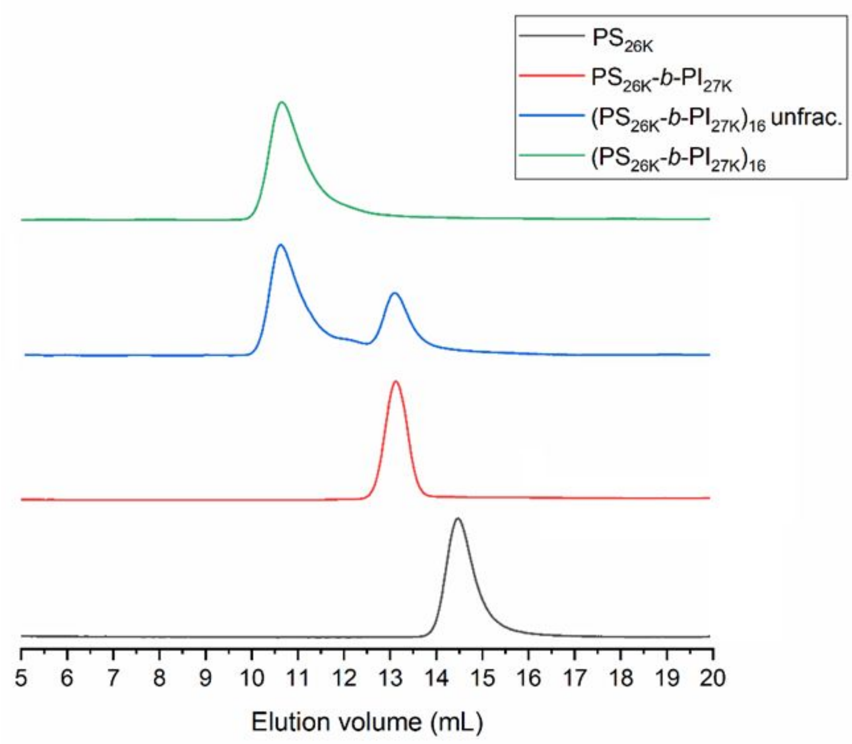

Figure S20. SEC traces of the PS (black line), PS-*b*-PI (red line), the unfractionated star block copolymer (blue line), and the final fractionated 16-arm star block copolymer (green line) in THF at 35 ^{0}C.

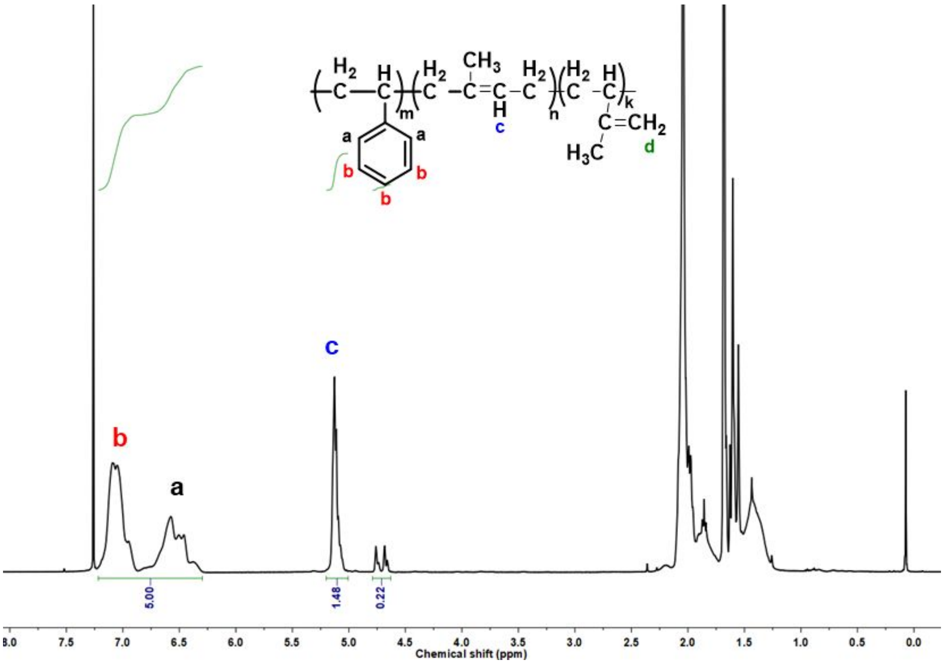

Figure S21. ^{1}H-NMR spectrum of the linear precursor, PS_{26K}-*b*-PI_{27K} (500 MHz, $CDCl_3$, 25 ^{0}C).

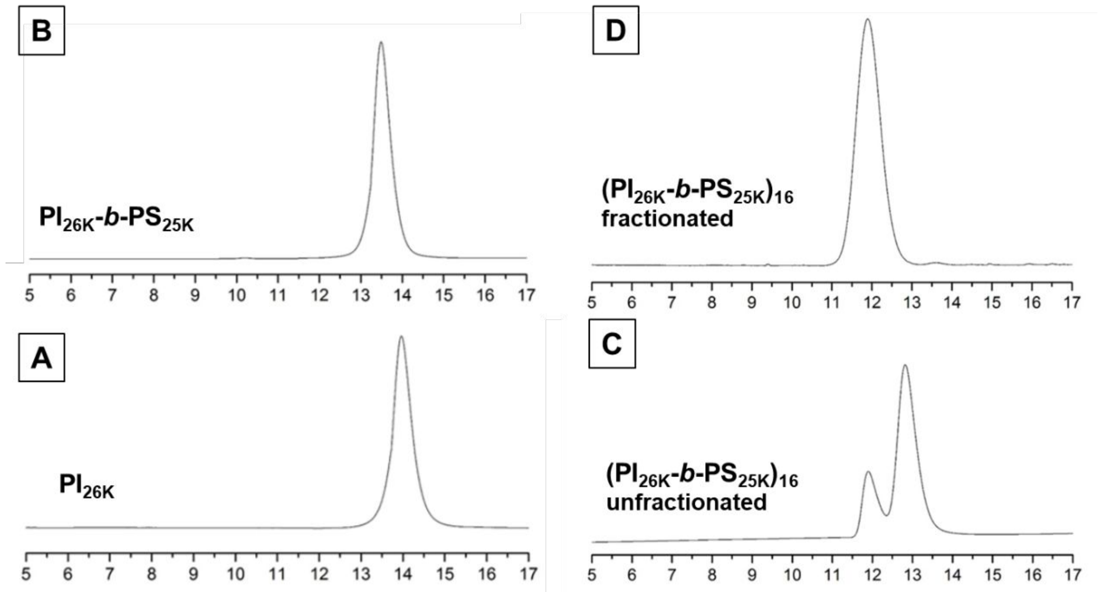

Figure S22. SEC traces of the (**A**) PI precursor, (**B**) PI-*b*-PS linear block copolymer, (**C**) unfractionated star block copolymer, and (**D**) final fractionated 16-arm star block copolymer [(PI-*b*-PS)$_{16}$] in THF at 35 ^{0}C.

.

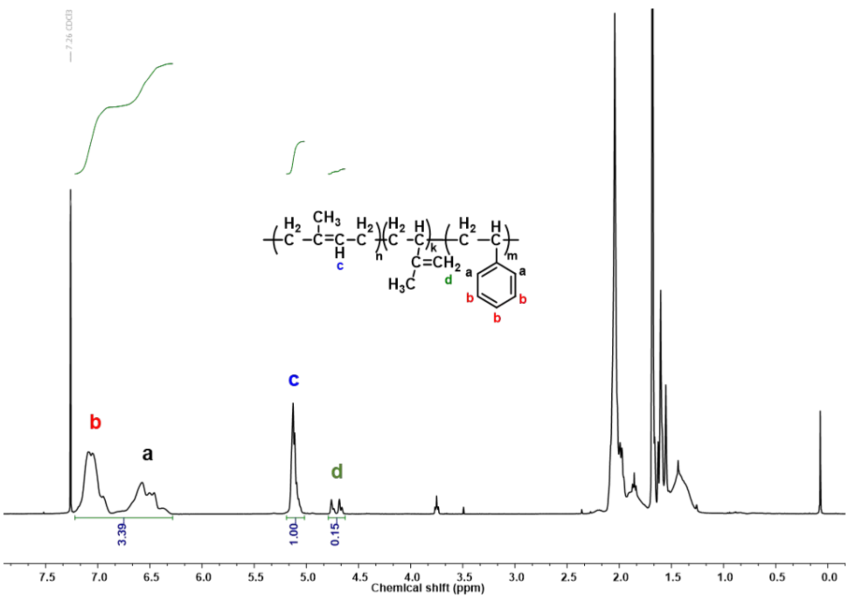

Figure S23. ^{1}H-NMR spectrum of the linear precursor PI_{26K}-*b*-PS_{25K} (500 MHz, $CDCl_3$, 25 ^{0}C).

.

References

(1) Rovigatti, L.; Capone, B.; Likos, C. N. Soft Self-Assembled Nanoparticles with Temperature-Dependent Properties. *Nanoscale* **2016**, *8* (6), 3288–3295.

(2) Davis, J. L.; Wang, X.; Bornani, K.; Hinestrosa, J. P.; Mays, J. W.; Kilbey, S. M. Solution Properties of Architecturally Complex Multiarm Star Diblock Copolymers in a Nonselective and Selective Solvent for the Inner Block. *Macromolecules* **2016**, *49* (6), 2288–2297.

(3) Moghimi, E.; Chubak, I.; Founta, D.; Ntetsikas, K.; Polymeropoulos, G.; Hadjichristidis, N.; Likos, C. N.; Vlassopoulos, D. The Influence of Arm Composition on the Self-Assembly of Low-Functionality Telechelic Star Polymers in Dilute Solutions. *Colloid and Polymer Science* **2021**, *299* (3), 497–507.

(4) Moghimi, E.; Chubak, I.; Statt, A.; Howard, M. P.; Founta, D.; Polymeropoulos, G.; Ntetsikas, K.; Hadjichristidis, N.; Panagiotopoulos, A. Z.; Likos, C. N. Self-Organization and Flow of Low-Functionality Telechelic Star Polymers with Varying Attraction. *ACS Macro Letters* **2019**, *8* (7), 766–772.

(5) Lu, B.; Laughlin, D. E.; Lambeth, D. N.; Wu, S. Z.; Ranjan, R.; Rauch, G. C. Texture Evolution in CoCrPtTa/Cr/NiAl Magnetic Recording Media. *Journal of Applied Physics* **1999**, *85* (8), 4295–4297.

(6) Fredrickson, G. H.; Bates, F. S. Dynamics of Block Copolymers: Theory and Experiment. *Annual Review of Materials Science* **1996**, *26* (1), 501–550.

(7) Ryu, C. Y.; Lee, M. S.; Hajduk, D. A.; Lodge, T. P. Structure and Viscoelasticity of Matched Asymmetric Diblock and Triblock Copolymers in the Cylinder and Sphere Microstructures. *Journal of Polymer Science Part B: Polymer Physics* **1997**, *35* (17), 2811–2823.

(8) Kossuth, M. B.; Morse, D. C.; Bates, F. S. Viscoelastic Behavior of Cubic Phases in Block Copolymer Melts. *Journal of Rheology* **1999**, *43* (1), 167–196.

(9) Sebastian, J. M.; Lai, C.; Graessley, W. W.; Register, R. A. Steady-Shear Rheology of Block Copolymer Melts and Concentrated Solutions: Disordering Stress in Body-Centered-Cubic Systems. *Macromolecules* **2002**, *35* (7), 2707–2713.

(10) Ekong, E. A.; Jayaraman, K. A Network Model for Melt Rheology of Block Copolymers. *Journal of Rheology* **1984**, *28* (1), 45–59.

(11) Doi, M.; Harden, J. L.; Ohta, T. Anomalous Rheological Behavior of Ordered Phases of Block Copolymers. 2. *Macromolecules* **1993**, *26* (18), 4935–4944.

(12) Spaans, R. D.; Williams, M. C. Nonlinear Viscoelasticity of ABA Block Copolymer Melts: Stress Relaxation and Recovery. *Industrial & Engineering Chemistry Research* **1995**, *34* (10), 3496–3507.

(13) Hashimoto, T.; Shibayama, M.; Kawai, H.; Watanabe, H.; Kotaka, T. Ordered Structure in Block Polymer Solutions. 2. Its Effect on Rheological Behavior. *Macromolecules* **1983**, *16* (3), 361–371.

(14) Watanabe, H.; Kotaka, T. Rheology of Styrene–Butadiene Diblock Copolymers Dissolved in Selective Solvent: Dependence on Molecular Dimension. *Polymer Journal* **1982**, *14* (9), 739–747.
(15) WATANABE, H.; KOTAKA, T. Rheology and Structure of Styrene-Butadiene Diblock Copolymers Dissolved in Selective Solvents. *Journal of Polymer Engineering* **1984**, *4* (1–2), 73–122.
(16) Henderson, C. P.; Williams, M. C. A Model for Triblock Copolymer Rheology. *Journal of Polymer Science: Polymer Letters Edition* **1979**, *17* (5), 257–261.
(17) Rubinstein, M.; Colby, R. H. *Polymer Physics*; Oxford university press New York, 2003; Vol. 23.
(18) Roos, A.; Creton, C. Effect of the Presence of Diblock Copolymer on the Nonlinear Elastic and Viscoelastic Properties of Elastomeric Triblock Copolymers. *Macromolecules* **2005**, *38* (18), 7807–7818.
(19) Hadjichristidis, N.; Iatrou, H.; Pispas, S.; Pitsikalis, M. Anionic Polymerization: High Vacuum Techniques. *Journal of Polymer Science Part A: Polymer Chemistry* **2000**, *38* (18), 3211–3234.
(20) Zhou, L. L.; Roovers, J. Synthesis of Novel Carbosilane Dendritic Macromolecules. *Macromolecules* **1993**, *26* (5), 963–968.
